# Tailoring the Structural Evolution of Multi‐Electron Redox Conversions via Strong Selenium–Carbon Interaction for Robust Aqueous Copper‐Ion Batteries

**DOI:** 10.1002/advs.202417084

**Published:** 2025-02-20

**Authors:** Fan Jiang, Haoyu Peng, Yiqian Wu, Yichen Li, Zeyu Zhang, Yue Wang, Jiuqiang Li, Jing Peng, Maolin Zhai

**Affiliations:** ^1^ Beijing National Laboratory for Molecular Sciences Radiochemistry and Radiation Chemistry Key Laboratory of Fundamental Science the Key Laboratory of Polymer Chemistry and Physics of the Ministry of Education College of Chemistry and Molecular Engineering Peking University Beijing 100871 P. R. China; ^2^ Chemical Defense Institute Beijing 100191 P. R. China

**Keywords:** aqueous batteries, conversion‐type cathodes, copper selenide

## Abstract

Aqueous metal‐selenium batteries based on chalcogenide cathodes, despite their multi‐electron conversion‐type redox reactions and rapid kinetics, suffer from short lifespans and unclear capacity degradation mechanisms. The interfacial interactions between doped carbon and chalcogenides correlate closely with the electrochemical structural evolution. Hence, flower‐like Cu_2−x_Se wrapped with ultrathin N‐doped carbon layer (Cu_2−x_Se@N‐C) is synthesized via a simple γ radiation‐pyrolysis route for the first time. The Cu_2−x_Se@N‐C cathode displays a high‐rate performance and long‐term stability, with a respective capacity of 310.6 mAh g^−1^ at 20 A g^−1^ and a capacity retention rate of 92.9% after 30 000 cycles over 2000 h at 5 A g^−1^. Ex situ X‐ray diffraction and X‐ray photoelectron spectroscopy confirm the reversible Cu storage mechanism of the Cu_2−x_Se@N‐C cathode and the issues of volume expansion and oxidative dissolution related to the capacity degradation of the Cu_2−x_Se cathode. Furthermore, X‐ray absorption analysis and theoretical calculations reveal the presence of Se─C interactions between the ultrathin N‐doped carbon and Cu_2−x_Se. As a result, the physical and chemical dual‐protection of N‐doped carbon via Se‐C not only effectively stabilizes the structural evolution of Cu_2−x_Se but also endows it with faster electrode reaction kinetics.

## Graphical abstract

The strong Se‐C interaction tailors the multi‐electron redox conversions of copper selenide, thereby inhibiting capacity degradation related to volume expansion and Se oxidation dissolution. Enhanced structural stability and rapid ion migration kinetics endow the flower‐like Cu_2–x_Se wrapped with ultrathin N‐doped carbon layer (Cu_2–x_Se@N‐C) with excellent long lifespan and high‐rate performance.

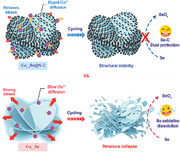

## 1 Introduction

Aqueous metal‐ion batteries attract extensive research interest because of their excellent safety, design flexibilities, low costs, and environmental friendliness.^[^ ^1^ ^]^ Among the various energy storage devices based on multivalent aqueous metal ions, metal‐sulfur (M‐S) battery systems involve multi‐electron phase‐conversion redox reactions with high theoretical capacities. This can significantly compensate for the energy density limitations of ion intercalation‐ and surface capacitance‐type aqueous metal batteries.^[^ ^2^ ^]^ However, S‐based cathode materials, such as S,^[^ ^3^ ^]^ CuS,^[^ ^4^ ^]^ and ZnS,^[^ ^5^ ^]^ generally suffer from severe levels of volumetric expansion, polysulfide dissolution, and poor conductivities.^[^ ^6^ ^]^ The sluggish electrode kinetics of the M‐S system restrict solid–solid conversion at high current densities,^[^ ^7^ ^]^ and the poor cycle stability caused by material fragmentation and detachment during cycling does not satisfy the durability requirements for practical applications.^[^ ^8^ ^]^

Se, owing to its excellent electrochemical activity and stability, may be the most promising candidate for replacing S for the fabrication of high‐performance M‐chalcogen batteries.^[^ ^9^ ^]^ Compared to that of S, Se exhibits a higher electrical conductivity (1 × 10^−3^ S m^−1^), which favors the maximal utilization of active materials and rapid electrode reactions.^[^ ^10^ ^]^ Current research demonstrated the viability of fabricating aqueous M‐Se battery systems. Zhi et al. developed a Zn‐Se battery system based on the solid‐phase‐conversion electrode reactions of Se and ZnSe.^[^ ^11^ ^]^ Two electron redox reactions endow Se‐based cathodes with an exceptionally high theoretical Zn^2+^ storage capacity (679.4 mAh g^−1^), yet still facing the serious issue of rapid capacity decay due to volume expansion and species dissolution. Compared to the Zn‐Se system, the Cu‐Se battery system displayed superior research potential and application prospects.^[^ ^12^ ^]^ Dai et al. used Cu ions as charge carriers to realize rapid, continuous four‐electron transfer reactions (Se ↔ CuSe ↔ Cu_3_Se_2_ ↔ Cu_2−x_Se ↔ Cu_2_Se) within a Se host.^[^ ^13^ ^]^ Cu ions act as charge carriers, exhibiting rapid insertion and extraction behaviors in the Se host to achieve excellent rate performance, maintaining a reversible capacity of 750 mAh g^−1^ even at 10 A g^−1^. Copper selenides with rich crystal structure diversity can trigger simultaneous valence changes at dual anionic‐cationic sites, inducing multi‐electron redox reactions with high theoretical specific capacities.^[^ ^14^ ^]^ Cubic Cu_2−x_Se is an excellent conversion‐type cathode material with an outstanding capacity to accommodate metal ions which could reversibly convert to CuSe in aqueous Cu‐ion batteries with a theoretical capacity of up to 378 mAh g^−1^.^[^ ^15^ ^]^ The non‐stoichiometric lattice structure of Cu_2−x_Se, with its abundant cationic vacancies, facilitates the rapid migration and storage of Cu^2+^ ions, thus exhibiting reversible Cu storage behaviors at high rates.^[^ ^16^ ^]^ In addition, Cu_2−x_Se exhibits an ultra‐high electrical conductivity as a metal‐like semiconductor, but the capacity fading mechanisms of aqueous Cu‐ion batteries have not been sufficiently studied. Wang et al. emphasized the impact of Cu_4_(SO_4_)(OH)_6_·H_2_O deposition induced by localized pH changes at the interface in CuSO_4_ electrolyte on cycling stability.^[^ ^17^ ^]^ To enhance the cycling stability and rate performance, high concentrations of H_2_SO_4_ was added for robust Cu‐Se battery. However, highly acidic electrolytes are not favorable for the application of practical battery system. Meanwhile, most studies have only reported the excellent stability of copper selenide cathode materials under high current densities, while neglecting the capacity degradation phenomenon that occurs under low current densities due to prolonged contact between the electrode and electrolyte during the charge–discharge process. Currently, the structural evolution of Cu_2−x_Se during cycling remains unclear, and effective modification strategies for fabricating aqueous Cu‐Se batteries with long‐term stabilities based on the Cu_2−x_Se cathode are lacking.

In this study, we synthesized an ultrathin N‐doped carbon‐wrapped flower‐like Cu_2−x_Se@N‐C composite via a simple two‐step γ radiation‐pyrolysis strategy. The special 3D morphology favored the exposure of the active sites during charging and discharging, and the N‐doped carbon layer effectively provided mechanical and chemical protection via Se─C bonds, enhancing the long‐term cycling stability at a high rate. Ex situ X‐ray diffraction (XRD) and X‐ray photoelectron spectroscopy (XPS) revealed the solid–solid conversion mechanisms of the Cu and Se redox sites with three electrons transfer redox reaction (Cu_2_Se ↔ Cu_2−x_Se ↔ Cu_3_Se_2_ ↔ CuSe). Additionally, the rapid capacity fading decay at low current densities is attributed to the oxidative Se precipitation and dissolution from the unstable structural evolution of Cu_2‐x_Se cathode. The strong Se‐C interaction between N‐doped carbon and Cu_2−x_Se on the surface of Cu_2−x_Se@N‐C effectively suppresses the Se‐containing active species loss caused by the frequent contact with the electrolyte, and significantly alleviates the volume expansion. The Cu_2−x_Se@N‐C cathode exhibited a remarkable capacity and negligible capacity decay of 310.6 mAh g^−1^ at 20 A g^−1^ and only 0.0002% per cycle for 30 000 cycles over 2000 h at 5 A g^−1^, respectively. This work delves into the structural evolution of Cu_2−x_Se cathodes in aqueous electrolytes and reveals the capacity degradation mechanisms, providing effective strategy to address the short cycling life‐span issue of conversion‐type electrode.

## 2 Results and Discussion

**Figure 1:**
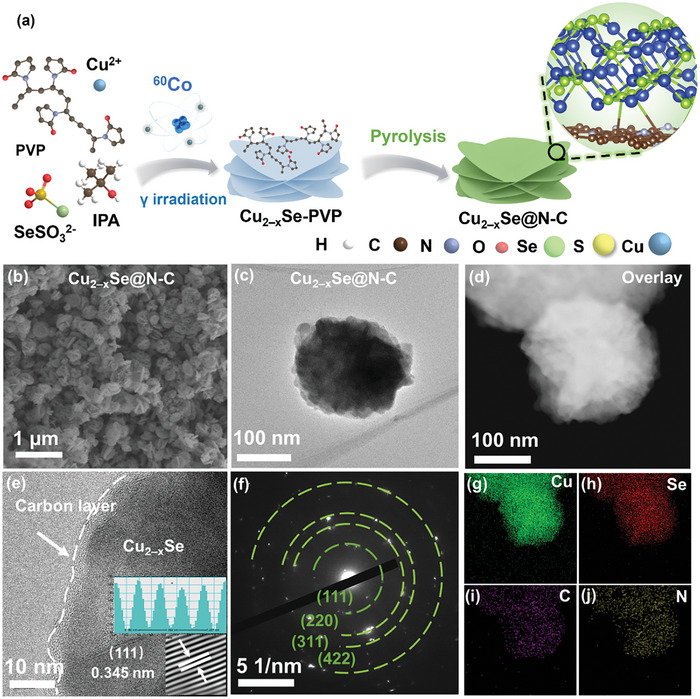
a) Schematic of the preparation of Cu_2−x_Se‐PVP and Cu_2−x_Se@N‐C. b) Scanning electron microscopy, c) transmission electron microscopy, and d) HAADF‐STEM images of Cu_2−x_Se@N‐C. e) HRTEM image and f) the corresponding SAED pattern of Cu_2−x_Se@N‐C. g–j) EDS maps of Cu_2−x_Se@N‐C. Ac, acetate; IPA, isopropyl alcohol.

**Figure** **1**a shows the two‐step process in synthesizing the N‐doped carbon‐coated Cu_2−x_Se composites. Initially, flower‐like Cu_2−x_Se‐polyvinylpyrrolidone (Cu_2−x_Se‐PVP) is prepared via γ‐radiation reduction.^[^ ^18^ ^]^ During radiation, the hydrated electrons produced via H_2_O radiolysis react with SeSO_3_^2−^ to form Se^2−^, which then slowly combines with the concurrently reduced Cu^+^ under the control of PVP to form Cu_2−x_Se crystal nuclei with abundant cationic defects. PVP adsorbed on the surface of Cu_2−x_Se confines the directional growth to flower‐shaped microspheres with approximate sizes of 100 nm (Figure S1, Supporting Information).^[^ ^19^ ^]^ However, the Cu_2−x_Se prepared without the addition of PVP, although exhibiting a morphology similar to that of Cu_2−x_Se‐PVP, has an overall size approaching 1 µm. The sharp diffraction signals of the cubic c‐Cu_2−x_Se (PDF#06‐0680) in the XRD pattern confirm the successful preparation of both Cu_2−x_Se and Cu_2−x_Se‐PVP using the radiation reduction method without impurities (Figure S2, Supporting Information).^[^ ^20^ ^]^ In subsequent calcination, PVP forms a N‐doped carbon layer in situ on the surface of copper selenide. The signal corresponding to g = 1.998 in the electron paramagnetic resonance (EPR) testing shown in Figure S3 (Supporting Information) is attributed to cation vacancies in the Cu_2−x_Se‐PVP structure, demonstrating the significant regulatory role of PVP in copper ion defects.^[^ ^21^ ^]^ Meanwhile, the concentration of copper ion defects in Cu_2−x_Se@N‐C only slightly decreases after calcination. The scanning electron microscopy (SEM) and high‐angle annular dark‐field scanning transmission electron microscopy (HAADF‐STEM) images shown in Figure 1b–d reveal that Cu_2−x_Se@N‐C retains its original flower‐like morphology without stacking or collapse after heating, owing to protection by PVP during carbonization. The high‐resolution transmission electron microscopy (HRTEM) images reveal that the C layer derived from PVP is tightly coated on the surface of the copper selenide with a thickness of only 3–5 nm, and no clear lattice fringes attributable to the carbon are observed (Figure 1e). This ultrathin carbon layer favors ion diffusion and the exposure of the redox‐active sites.^[^ ^22^ ^]^ The lattice fringe with an interplanar spacing of 0.345 nm is attributed to the (111) plane of cubic Cu_2‐x_Se.^[^ ^23^ ^]^ The polycrystalline diffraction rings in the selected‐area electron diffraction (SAED) pattern shown in Figure 1f are consistent with the (111), (220), (311), and (422) planes, indicating that the copper selenide displays a high crystallinity. The homogeneous distributions of N and C throughout the sample, as indicated by the results of energy‐dispersive X‐ray spectroscopy (EDS), confirm the uniform wrapping of N‐doped carbon on Cu_2−x_Se (Figure 1g–j).

**Figure 2:**
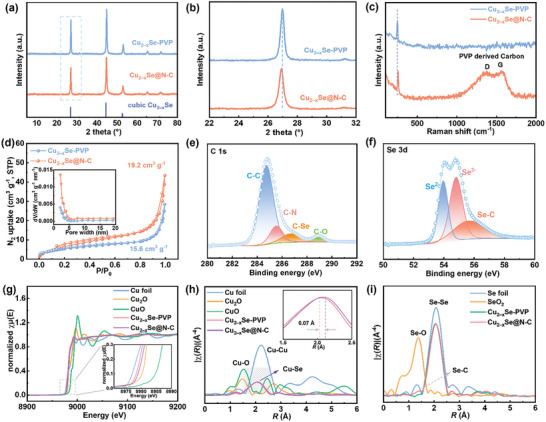
a) XRD patterns of Cu_2−x_Se‐PVP and Cu_2−x_Se@N‐C. b) Partial enlargement of the regions of the XRD patterns representing the (111) planes. c) Raman spectra, d) N_2_ adsorption–desorption isotherms (inset shows the corresponding pore size distributions) of Cu_2−x_Se‐PVP and Cu_2−x_Se@N‐C e,f) High‐resolution e) C 1s and f) Se 3d XPS spectra of Cu_2−x_Se@N‐C. g) Cu K‐edge XANES spectra of Cu_2−x_Se‐PVP and Cu_2−x_Se@N‐C and h) K^3^‐weighted χ(*R*)‐functions of the EXAFS spectra (the inset shows a partial enlargement). i) Se K^3^‐weighted χ(*R*)‐functions of the EXAFS spectra of Cu_2−x_Se‐PVP and Cu_2−x_Se@N‐C.

Further investigation of the structural changes before and after calcination was conducted using XRD. After calcination, the crystal phase of Cu_2−x_Se@N‐C does not change significantly, and no other species are observed, with the cubic c‐Cu_2−x_Se structure retained (**Figure** **2**a). However, the diffraction peaks representing various crystal planes shift slightly to lower angles, with the diffraction peak representing the (111) plane shifting by 0.2° (Figure 2b). This suggests that the carbonization of PVP leads to the lattice expansion of Cu_2−x_Se. This favors the rapid insertion and release of Cu ions within bulk copper selenide.^[^ ^24^ ^]^ At higher calcination temperatures, new diffraction peaks attributed to Cu_3_Se_2_ are observed in the XRD pattern, indicating that a phase transition from Cu_2−x_Se to Cu_3_Se_2_ occurs within Cu_2−x_Se@N‐C‐600 (Figure S4, Supporting Information).^[^ ^25^ ^]^ In the Raman spectrum of Cu_2−x_Se‐PVP (Figure 2c), the peak at 258.6 cm^−1^, which is attributed to the Cu‐Se structure, shifts to 283.6 cm^−1^ after pyrolysis.^[^ ^26^ ^]^ Therefore, the lattice expansion within Cu_2−x_Se@N‐C affects the vibrational modes of Cu‐Se within the lattice, resulting in the weakened intensities of the Cu‐Se vibrations. Additionally, the D and G peaks of the C structure are observed in the range of 1300–1600 cm^−1^ with an intensity ratio (I_D_/I_G_) of 1.02, indicating that the N‐doped carbon layers of Cu_2−x_Se@N‐C are highly amorphous.^[^ ^27^ ^]^ This further confirms the successful carbonization of PVP on the surface of Cu_2−x_Se. Moreover, the I_D_/I_G_ of the carbon species gradually decreases with increasing pyrolysis temperature, suggesting the enhanced order of the carbon layer (Figure S5, Supporting Information). The Cu_2−x_Se contents of the samples were calculated using thermogravimetric analysis in air. Figure S6 (Supporting Information) shows that the residual PVP molecules on the surface of Cu_2−x_Se‐PVP begin to decompose and release CO_2_ with the assistance of O_2_ as the temperature rises to 250 °C.^[^ ^19^ ^]^ When the temperature increases to 350 °C, Cu_2−x_Se begins to oxidize to CuO and SeO_2_ leading to a weight increase. After heating to 600 °C, the SeO_2_ species vaporize, leaving CuO as the final product. Compared to that of Cu_2−x_Se‐PVP, Cu_2−x_Se@N‐C exhibits a smaller mass loss, which can be attributed to the preliminary decomposition of the surface PVP via carbonization.^[^ ^28^ ^]^ The mass percentage of Cu_2−x_Se within Cu_2−x_Se@N‐C is 90.2 wt.%, and Cu_2−x_Se@N‐C‐400 and Cu_2−x_Se@N‐C‐600 exhibit similar contents (90.5 and 91.9 wt.%, respectively, Figure S7, Supporting Information). Elemental analysis and inductively coupled plasma atomic emission spectroscopy were employed to determine the N content of the doped C layer and the Cu/Se atomic ratio of Cu_2−x_Se. The mass percentage of N within Cu_2−x_Se@N‐C is 1.6 wt.%, with a Cu:Se atomic ratio of 1.73:1. The specific surface areas and pore size distributions of the samples were determined using isothermal N_2_ adsorption–desorption (Figure 2d). The specific surface area of the flower‐like Cu_2−x_Se‐PVP is 15.6 cm^3^ g^−1^, which increases to 19.2 cm^3^ g^−1^ owing to the mesoporous structures derived from carbonized PVP on the surface of Cu_2−x_Se@N‐C. The increased surface area facilitates thorough interfacial contact and accessibility for ion migration. However, the inadequate carbonization of Cu_2−x_Se@N‐C‐400 at lower temperatures and the structural collapse caused by the phase transition within Cu_2−x_Se@N‐C‐600 reduce the specific surface area and significantly decrease the number and volume of pores (Figure S8, Supporting Information). The chemical environments and coordination structures of the elements within Cu_2−x_Se@N‐C were characterized using XPS (Figures S9 and S10, Supporting Information). In the high‐resolution C 1s spectrum, in addition to the signals representing C─C, C─N, and C─O, a characteristic peak is observed at 286.8 eV, corresponding to the C─Se bonds (Figure 2e).^[^ ^29^ ^]^ This indicates strong interactions at the interface between the N‐doped carbon layer and Cu_2−x_Se.^[^ ^30^ ^]^ The high‐resolution N 1s spectrum shows that the N species mainly occur as pyridinic (398.7 eV) and pyrrolic N (400.5 eV), with a small amount present as graphite N (401.8 eV, Figure S11, Supporting Information).^[^ ^31^ ^]^ The similar distributions of N species within Cu_2−x_Se@N‐C‐400 and Cu_2−x_Se@N‐C‐600 indicate that the pyrolysis temperature has a weaker interference effect on N doping (Figure S12, Supporting Information). The Cu within Cu_2−x_Se mainly occurs as Cu^+^ (932.3 eV), with a small amount of Cu^2+^ (933.4 eV), which is consistent with the rich cationic defects of the non‐stoichiometric Cu_2−x_Se structure,^[^ ^32^ ^]^ corroborating the results of XRD and EPR. The presence of high‐valent Cu_3_Se_2_, owing to the phase transition at high temperatures, increases the proportion of Cu^2+^ species (Figure S13, Supporting Information). The peaks at 53.9 and 54.8 eV in the high‐resolution Se 3d spectrum correspond to Se^2−^ 3d_3/2_ and Se^2−^ 3d_5/2_, respectively (Figure 2f).^[^ ^33^ ^]^ Moreover, the signal corresponding to the Se─C structure is observed at 55.6 eV, again confirming the strong interaction between the ultrathin carbon layer and Cu_2−x_Se. At different pyrolysis temperatures, the C 1s and Se 3d spectra display similar characteristic peaks attributed to the Se─C structure. Synchrotron X‐ray absorption was used to further reveal the differences in the valence states and lattice coordination environments within the samples. The Cu K‐edge X‐ray absorption near‐edge structure (XANES) spectra of Cu_2−x_Se‐PVP and Cu_2−x_Se@N‐C are similar (Figure 2g), with white line positions situated between those of Cu foil and Cu_2_O, indicating that the Cu valences within the samples range from 0 to +1. Because of the strong electron‐donating effect of N‐doped carbon with higher charge densities at the Cu sites, the white line position of Cu_2−x_Se@N‐C is considerably closer to that of Cu foil. The characteristic peaks at 2.02 and 2.09 Å in the related K^3^‐weighted χ(*R*)‐functions of the extended X‐ray absorption fine structure (EXAFS) spectra shown in Figure 2h are attributed to the Cu─Se bonds within Cu_2−x_Se‐PVP and Cu_2−x_Se@N‐C, respectively. Additionally, Cu─O and Cu─Cu bonds are not detected.^[^ ^34^ ^]^ This confirms the successful synthesis of Cu_2−x_Se using γ radiation and the lattice expansion after pyrolysis. Furthermore, the Se K‐edge XANES spectra confirm the charge transfer behavior close to the Se─C structure, with the Se valence state within Cu_2−x_Se@N‐C closer to (Figure S14, Supporting Information). The Se K^3^‐weighted χ(*R*)‐functions of the EXAFS spectra also exhibit similar curve shapes (Figure 2i), but the stronger signal of Cu_2−x_Se@N‐C at 1.41 Å is attributed to Se─C,^[^ ^35^ ^]^ indicating the presence of the strong interaction between the N‐doped carbon and Cu_2−x_Se.

**Figure 3:**
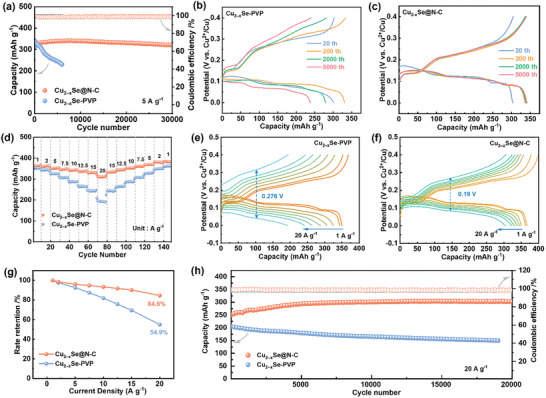
a) Long‐term cycling stabilities of Cu_2−x_Se‐PVP and Cu_2−x_Se@N‐C at 5.0 A g^−1^. b,c) GCD profiles of b) Cu_2−x_Se‐PVP and c) Cu_2−x_Se@N‐C at various cycle numbers. d) Rate capabilities of Cu_2−x_Se‐PVP and Cu_2−x_Se@N‐C. e,f) GCD profiles of e) Cu_2−x_Se‐PVP and f) Cu_2−x_Se@N‐C based on various current densities. g) rate retention of Cu_2−x_Se‐PVP and Cu_2−x_Se@N‐C. h) Cycling performances of Cu_2−x_Se‐PVP and Cu_2−x_Se@N‐C at 20.0 A g^−1^.

Aqueous Cu‐ion coin batteries were assembled for electrochemical performance evaluation. The Cu_2‐x_Se‐PVP synthesized using γ radiation with the control of PVP exhibits a smaller flower‐like nanostructure. The 2D nanosheets enable sufficient contact with the electrolyte, which favors the intercalation and extraction of the Cu^2+^ within the Cu_2−x_Se host, thus enhancing the accessible capacity (Figure S15, Supporting Information). The initial discharge capacities of Cu_2−x_Se‐PVP and Cu_2−x_Se@N‐C at 5 A g^−1^ are 79.2 and 79.0 mAh g^−1^, respectively, exhibiting similar discharge behaviors (Figure S16, Supporting Information). However, the galvanostatic charge–discharge (GCD) curves indicate that the specific capacity of Cu_2−x_Se‐PVP is up to 318.5 mAh g^−1^ in the second discharge cycle with a plateau potential of 0.122 V and gradually increases from the third cycle onward. In contrast, Cu_2−x_Se@N‐C exhibits a discharge capacity of 312.6 mAh g^−1^ with a slightly higher plateau potential than that of Cu_2−x_Se‐PVP (0.132 V), with a slowly increasing trend. The initial capacity differences and variation patterns are due to the fragmentation and detachment of the active material during the phase conversion of Cu_2−x_Se, while the volume expansion of N‐doped carbon‐layer‐coated Cu_2−x_Se@N‐C is effectively mitigated. **Figure** **3**a shows that the discharge capacity of Cu_2−x_Se‐PVP rapidly decays, with a reversible capacity of only 230.3 mAh g^−1^ after 6000 cycles, corresponding to a capacity retention of 68.8%. Meanwhile, the reversible capacity of Cu_2−x_Se@N‐C can still reach 317.4 mAh g^−1^ after 30 000 cycles over 2000 h, with a negligible capacity decay of 0.0002% per cycle (based on the maximum discharge capacity of 341.3 mAh g^−1^), and the Coulombic efficiency remains at ∼99%. Moreover, the capacity retention rates of Cu_2−x_Se@N‐C‐400 and Cu_2‐x_Se@N‐C‐600 are 91.3% and 81.1% at 5 A g^−1^, respectively. Although Cu_2‐x_Se@N‐C‐400 exhibits good cycling stability, its peak reversible specific capacity (321.4 mAh g^−1^) is considerably lower than that of Cu_2−x_Se@N‐C (Figure S17, Supporting Information). The GCD curves of Cu_2−x_Se@N‐C virtually overlap upon long‐term cycling, further indicating its excellent cycling stability, but the discharge plateau potential of Cu_2−x_Se‐PVP decreases to 0.07 V (Figure 3b,c) after the 5000^th^ cycle. As cycling progresses, the Cu_2−x_Se‐PVP and Cu_2−x_Se@N‐C electrode continuously undergoes irreversible pulverization, enhancing its contact with the electrolyte and promoting the accessibility of ions at the electrode interface, thus leading to a continuous increase in reversible capacity in the early stages of cycling.^[^ ^36^ ^]^ For Cu_2−x_Se@N‐C, the buffering effect of the carbon layer significantly slows down the occurrence of fracturing, resulting in a more gradual increase in reversible capacity. More significantly, Cu_2−x_Se@N‐C exhibits superior high‐rate performance compared to that of Cu_2‐x_Se‐PVP. Figure 3d shows that the reversible capacities of Cu_2−x_Se@N‐C at current densities of 1, 2, 5, 7.5, 10, 12.5, 15, and 20 A g^−1^ are 361.4, 358.4, 348.6, 344.5, 340.9, 335.6, 331.2, and 310.6 mAh g^−1^, respectively. However, the reversible capacity of Cu_2−x_Se‐PVP can only reach 193.4 mAh g^−1^ at 20 A g^−1^, with a capacity retention rate of 55.3%. The GCD profiles at different current densities reveal that Cu_2−x_Se‐PVP exhibits severe polarization at high current densities, corresponding to a difference of 0.276 V in the charge–discharge plateau potential. However, the GCD profiles of Cu_2−x_Se@N‐C do not change significantly as the current density increases, and the difference in polarization potential is only 0.190 V, indicating rapid reaction kinetics (Figure 3e,f). At a high current density of 20 A g^−1^, the rate retention is as high as 84.6% (relative to that at 1 A g^−1^, Figure 3g). This is because of the porous ultrathin N‐doped carbon layer, which not only enhances charge transfer between the active particles but also facilitates effective electrolyte infiltration.^[^ ^37^ ^]^ Compared to those of Cu_2−x_Se@N‐C, Cu_2−x_Se@N‐C‐400 and Cu_2−x_Se@N‐C‐600 display unsatisfactory Cu storage capacities at high current densities, with respective retention rates of 66.8% and 76.5% at 20 A g^−1^ (Figure S18, Supporting Information). Even at an ultra‐high current density of 20 A g^−1^, Cu_2−x_Se@N‐C can operate stably with a Coulombic efficiency of almost 100%. Cu_2−x_Se@N‐C can still exhibit a high discharge capacity of 302.8 mAh g^−1^ after 20000 cycles, far superior to Cu_2−x_Se‐PVP (Figure 3h).

**Figure 4:**
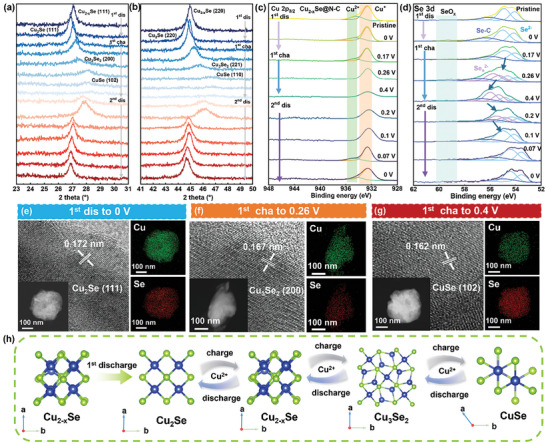
a,b) Ex situ XRD patterns of the Cu_2−x_Se@N‐C cathode during the 1st cycle and 2nd discharge. c,d) Ex situ c) Cu 2p_3/2_ and d) Se 3d XPS spectra of the Cu_2−x_Se@N‐C cathode during the 1st cycle and 2nd discharge. e–g) HRTEM and EDS images of Cu_2−x_Se@N‐C in various states (insets show the corresponding HAADF‐STEM images). h) Schematic of the conversion mechanism of the Cu_2−x_Se@N‐C cathode.

Ex situ XRD was conducted to reveal the Cu‐ion storage mechanism of Cu_2−x_Se@N‐C during the 1st cycle and 2nd discharge (**Figure** **4**a,b; Figure S19, Supporting Information). Initially, Cu_2−x_Se@N‐C exhibits the typical cubic structure of Cu_2−x_Se. As the 1st discharge proceeds, the diffraction peaks representing the (111) and (220) planes of Cu_2−x_Se shift to lower angles, indicating continuous lattice expansion initiated by the diffusion and intercalation of Cu ions into the Cu_2−x_Se bulk phase. Upon discharging to 0 V, Cu_2−x_Se is completely transformed into cubic Cu_2_Se. During the first charge to 0.17 V, the diffraction peak of Cu_2−x_Se (111) plane returns to its initial position, indicating that Cu ions are extracted from the Cu_2_Se structure, leaving rich cationic defects. As charging continues, the diffraction peak of Cu_2−x_Se (111) plane gradually disappears, and weaker diffraction peaks corresponding to Cu_3_Se_2_ (200) and (221) are observed. When fully charged to 0.4 V, only the peaks representing the (102) and (110) planes of CuSe are observed in the XRD pattern of Cu_2−x_Se@N‐C, suggesting that Cu_2−x_Se@N‐C is completely converted to CuSe in the final charged state. During the 2nd discharge, the XRD patterns reveal that Cu_2−x_Se@N‐C can be completely reconstructed from CuSe to Cu_2_Se via the same multiple‐phase‐transition behaviors. When discharged to 0 V, no residual intermediate‐phase diffraction peaks are observed and the diffraction peak representing Cu_2_Se (111) is restored to a position similar to that observed during the 1st discharge. These results demonstrate the high reversibility of the Cu storage behavior of the Cu_2−x_Se@N‐C cathode.^[^ ^38^ ^]^ The changes in the valence states of Cu_2−x_Se@N‐C were investigated further using ex situ XPS to understand the role of the N‐doped carbon layer (Figure 4c,d). Initially, the characteristic peaks at 932.7 and 934.8 eV in the Cu 2p_3/2_ spectrum are respectively attributed to the Cu^+^ and Cu^2+^ species within Cu_2−x_Se. After completing 1st discharge process, the peak representing Cu^2+^ weakens, confirming that the Cu^2+^ within Cu_2−x_Se@N‐C is reduced to Cu^+^. During the 1st charge, the signal representing Cu^+^ also gradually weakens, and that of Cu^2+^ is not observed. After charging to 0.26 V, the characteristic peak of Cu^+^ shifts to a lower binding energy owing to oxidative dissolution as Cu^2+^ is extracted from Cu_2_Se and dissolved in the electrolyte, thus weakening the intensity of the Cu^+^ signal. As the complex phase transitions proceed upon the release of Cu^2+^, the coordination environment of Se around Cu changes, leading to a red shift in the binding energy. When the potential is discharged back to 0 V, the peak of Cu^+^ is restored to its initial state without significant changes, indicating that the Cu^2+^ in the electrolyte can be reversibly stored within the Cu_2−x_Se@N‐C cathode. Moreover, this analysis confirms that the Cu_2−x_Se within Cu_2−x_Se@N‐C exhibits Cu^2+^/Cu^+^ redox couples as the cationic active centers. In the high‐resolution Se 3d spectrum, the Se within Cu_2−x_Se is initially observed as a Se^2−^ doublet. During the 1st discharge and subsequent charge to 0.17 V, the same characteristic peaks of Se^2−^ are observed. No changes are observed in the valence state of Se during the structural transition between Cu_2−x_Se and Cu_2_Se. As the charging potential increases from 0.17 to 0.4 V, the intensities of the characteristic peaks of Se^2−^ weaken, and new peaks attributed to Se_n_^2−^ are observed at higher binding energies. The emergence of the Se_n_^2−^ species is due to the oxidative coupling of Se^2−^. After discharging to 0.07 V, the Se_n_^2−^ species are no longer detected, and the Se^2−^peaks return to their initial states, consistent with Cu 2p_3/2_. This confirms the high reversibility of the redox conversion between the Se_n_^2−^ and Se^2−^ species. Furthermore, the valence state of the Se within Cu_2−x_Se can switch with the insertion/release of Cu ions, acting as extra redox sites. The Cu/Se dual sites redox reactions were also corroborated by ex situ Raman experiments, as shown in Figure S20 (Supporting Information). The signal attributed to Cu‐Se located at 283.6 cm^−1^ in Cu_2−x_Se@N‐C did not show significant changes during the charge and discharge process, indicating the structural stability of copper selenide within the transformation. Meanwhile, as the 1st charging process proceeded, a Se‐Se vibration signal corresponding to the intermediate Cu_3_Se_2_ and the final charged product CuSe appears at a lower Raman shift of 262.9 cm^−1^.^[^ ^39^ ^]^ This Se‐Se vibration signal can reversibly disappear during the subsequent 2nd discharge process, further revealing the anionic energy storage mechanism at the Se sites. More importantly, compared to Cu_2−x_Se‐PVP, Cu_2−x_Se@N‐C can undergo continuous conversion reactions under lower potential polarization, enabling rapid multi‐electron redox reactions between Cu_2_Se and CuSe. Ex situ TEM was used to further monitor the various intermediate products during the charging and discharging processes. The lattice spacings of 0.172, 0.167, and 0.162 nm exhibited in Figure 4e–g  are attributed to the planes of Cu_2_Se (111), Cu_3_Se_2_ (200), and CuSe (102). Additionally, the results of EDS indicate that the semi‐quantitative Cu/Se ratios are close to the theoretical stoichiometric compositions of the intermediates of the different charge–discharge processes, consistent with the aforementioned ex situ XRD results. In summary, the energy storage mechanism of Cu_2−x_Se@N‐C can be divided into two parts (Figure 4h). During charging, Cu_2_Se first transforms into Cu_2−x_Se as Cu^+^ oxidized to Cu^2+^ and dissolves into electrolyte, with only the Cu valence state changing while the Se element valence state remains constant. As Cu^2+^ continues to be released from Cu_2−x_Se, the copper selenide undergoes continuous structural transitions from Cu_2−x_Se to Cu_3_Se_2_ and then to CuSe. During these multi‐electron transfer steps, the continuous increases in valences of the Cu ions simultaneously induce the reaction of Se in the oxidation reaction. During discharging, the Se species and Cu^2+^ are reduced to their initial valence states, transitioning via CuSe, Cu_3_Se_2_, and Cu_2−x_Se and to fully discharged product Cu_2_Se. However, Cu_2−x_Se‐PVP exhibits a different storage behavior from that of Cu_2−x_Se@N‐C (Figure S21, Supporting Information). The 1st discharge product Cu_2_Se, exhibits an anomalous signal representing Cu^2+^, which gradually disappears during the subsequent charging from Cu_2_Se to Cu_2‐x_Se. When the Cu_2−x_Se‐PVP cathode is discharged from 0.4 to 0.1 V, the Cu^+^ species remains consistent and no signal of Cu^2+^ is observed. However, after discharging to 0.07 V, the signal of Cu^2+^ is observed again as the intensity increases and potential decreases. To determine the cause behind the presence of the Cu^2+^ species, the Se 3d spectrum of Cu_2−x_Se‐PVP was further analyzed. Upon the 1st discharge to 0 V, the characteristic peak representing Se^2−^ does not change significantly compared to that of the initial state, but a new peak attributed to high‐valence SeO_x_ species is observed at 59.4 eV.^[^ ^40^ ^]^ The signal representing SeO_x_ gradually weakens during the subsequent charge, and it is no longer observed after charging to 0.26 V. However, SeO_x_ is again detected upon conversion from Cu_2−x_Se to Cu_2_Se during the 2nd discharge, with the corresponding intensity increasing upon further discharge. The SeO_x_ species exhibits a consistent periodic appearance with high‐valence Cu^2+^ during the conversion between Cu_2−x_Se and Cu_2_Se. Therefore, during discharging, Se may be oxidized to form SeO_x_^2−^ and combine with Cu^2+^ from the electrolyte to form insoluble CuSeO_x_, which may be deposited on the surface of the active material.^[^ ^41^ ^]^ During charging, the CuSeO_x_ precipitate is further oxidized and dissolves to form Cu^2+^ and SeO_4_^2−^, which are immediately released into the electrolyte. The oxidative dissolution of Se during charging/discharging is the main cause of the capacity decay of Cu_2−x_Se‐PVP. In contrast, the N‐doped carbon layer‐encapsulated Cu_2−x_Se@N‐C does not exhibit a clear Se dissolution behavior. Inductively coupled plasma mass spectrometry was further utilized to monitor the concentration of selenide species dissolved in the electrolyte during this charge–discharge process (Figure S22, Supporting Information). Initially, Cu_2−x_Se‐PVP exhibits severe dissolution, with the Se content in the solution slowly increasing during the 1st discharge and subsequent charging until conversion to Cu_2−x_Se. However, the Se content increases significantly after the reaction of Cu_2−x_Se with Cu_3_Se_2_, which is related to the oxidation of the selenium oxides on the surface of Cu_2−x_Se‐PVP to soluble SeO_4_^2−^. When discharged back to Cu_2−x_Se and Cu_2_Se, several SeO_4_^2−^ species from the solution are reduced to form insoluble copper selenate. The protective effect of N‐doped carbon ensures the structural stability of Cu_2−x_Se@N‐C in a 0.5 m CuSO_4_ solution, with only a slight increase in the Se content accompanying the redox reaction.^[^ ^42^ ^]^

**Figure 5:**
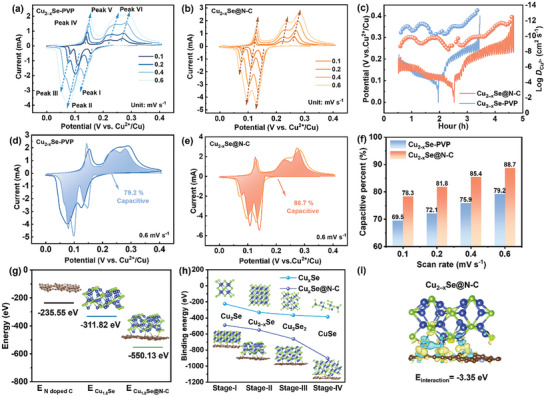
a,b) Cyclic voltammograms of a) Cu_2−x_Se‐PVP and b) Cu_2−x_Se@N‐C, as obtained at various scan rates from 0.1 to 0.6 mV s^−1^. c) *D*_Cu_^2+^ values of Cu_2−x_Se‐PVP and Cu_2−x_Se@N‐C, as calculated using the GITT. d,e) Current contributions of d) Cu_2−x_Se‐PVP and e) Cu_2−x_Se@N‐C at 0.6 mV s^−1^. f) Capacitive current ratios of Cu_2−x_Se‐PVP and Cu_2−x_Se@N‐C at various scan rates. g) Optimized model and the corresponding binding energy between Cu_2−x_Se and N‐doped carbon. h) Schematic of the binding energies of Cu_x_Se and Cu_x_Se@N‐C along the reaction pathway. i) Differential charge density of the Cu_2‐x_Se@N‐C heterointerface.

The electrochemical storage behaviors of Cu_2−x_Se‐PVP and Cu_2−x_Se@N‐C were investigated using a two‐electrode test system. The cyclic voltammograms of Cu_2−x_Se‐PVP and Cu_2−x_Se@N‐C obtained at different scan rates (0.1, 0.2, 0.4, and 0.6 mV s^−1^) are shown in **Figure** **5**a,b, respectively. The cyclic voltammograms of Cu_2−x_Se‐PVP and Cu_2−x_Se@N‐C display similar shapes, including three pairs of redox peaks, which represent energy storage behaviors based on multistep electron transfer. Notably, the redox current peak of Cu_2−x_Se@N‐C does not change significantly with increasing scan rate, indicating that the potential polarization is considerably weaker than that of Cu_2−x_Se‐PVP.^[^ ^43^ ^]^ Hence, Cu_2−x_Se@N‐C exhibits faster electrode reaction kinetics and a high rate performance. The Cu‐ion diffusion coefficient *D*_Cu_^2+^ was calculated using the galvanostatic intermittent titration technique (GITT).^[^ ^44^ ^]^ Figure 5c shows that Cu_2−x_Se@N‐C exhibits a longer charge–discharge time than that of Cu_2−x_Se‐PVP at 0.5 A g^−1^, suggesting that Cu_2−x_Se@N‐C displays a superior Cu storage capacity. The *D*_Cu_^2+^ of the Cu_2−x_Se@N‐C cathode in 0.5 M CuSO_4_ is in the range 10^−8^–10^−13^, far exceeding that of Cu_2−x_Se‐PVP (10^−9^–10^−14^). This is mainly attributed to the optimization of the ion transport and interfacial contact provided by the ultrathin porous N‐doped carbon, in addition to the lattice expansion induced during pyrolysis, which provides effective channels for rapid ion migration. The results of electrochemical impedance spectroscopy (EIS) indicate that the charge transfer resistances *R*_ct_ of Cu_2−x_Se‐PVP and Cu_2−x_Se@N‐C are 5.6 and 4.3 Ω, respectively (Figure S23, Supporting Information). This confirms that the N‐doped carbon layer enhances the charge transfer capacity of the surface via the Se─C bonds, which is favorable for improving the chemical reaction kinetics. In the EIS spectrum, the slope in the low‐frequency region reflects the Warburg impedance related to the ion transport capacity.^[^ ^45^ ^]^ The lower slope of Cu_2−x_Se@N‐C indicates its superior Cu‐ion diffusion kinetics, which are consistent with the results of the GITT. Meanwhile, the *R*_ct_ values of Cu_2−x_Se@N‐C‐400 and Cu_2−x_Se@N‐C‐600 are 5.8 and 8.7 Ω, respectively, suggesting that their suboptimal rate performances are mainly due to their increased charge transfer impedances owing to insufficient carbonization and phase transitions (Figure S24, Supporting Information). The *R*_ct_ values were also monitored at different cycle numbers at 5 A g^−1^ (Figure S25, Supporting Information), and the *R*_ct_ of Cu_2−x_Se‐PVP increases rapidly with cycling, reaching 19.8 and 62.2 Ω after 200 and 2000 cycles, respectively. In contrast, the *R*_ct_ of Cu_2−x_Se@N‐C does not change significantly during the first 2000 cycles, and it only increases to 13.4 Ω after 5000 cycles. SEM images (Figure S26, Supporting Information) reveal that Cu_2−x_Se‐PVP experiences severe agglomeration and fragmentation after extended cycling (5000 cycles), and the detachment of the active material and poor contact with the electrolyte significantly increase the *R*_ct_ value, leading to rapid capacity decay. However, the flower‐like microsphere structure of the Cu_2−x_Se@N‐C cathode remains clear and well‐dispersed, confirming the crucial protective role of the ultrathin N‐doped carbon layer against volume expansion during charging‐discharging. Monitoring the content of dissolved Se within the electrolyte after extended cycling indicates that severe Se dissolution occurs after 2000 cycles using Cu_2−x_Se‐PVP, whereas Cu_2−x_Se@N‐C can maintain a lower Se concentration (Figure S27, Supporting Information). This suggests that N‐doped carbon can minimize problems related to chemical oxidative dissolution via Se─C interactions, further enhancing the structural stability. To further investigate the Cu storage behaviors of the materials, the peak currents (*i*) of the various redox peaks in the cyclic voltammograms obtained at different scan rates are fitted using Equation (1)^[^ ^46^ ^]^:

(1) $$i=av^{b}$$

Here, a and b are the parameters of the energy storage process. When b = 0.5, ion diffusion is the main component of the electrochemical process,^[^ ^47^ ^]^ whereas the pseudocapacitive behavior controls the electrochemical process when b approaches 1. The relationship between log(*i*) and log(*v*) reveals that the b values of the three oxidation peaks (Peaks I, II, and III) of Cu_2−x_Se@N‐C are 0.77, 0.87, and 0.92, respectively, whereas the b values of the reduction peaks (Peaks IV, V, and VI) are 0.89, 0.7, and 0.88, respectively (Figure S28, Supporting Information). Therefore, the ion diffusion and pseudocapacitive processes jointly control the electrochemical behavior of Cu_2−x_Se@N‐C. The fitting results of Cu_2−x_Se‐PVP (Figure S29, Supporting Information) also indicate a mixed electrochemical behavior, with a lower proportion of pseudocapacitive processes. The electrochemical behavior is further evaluated by assessing the proportion of the pseudocapacitive‐controlled current in the total charge–discharge current using Equation (2)^[^ ^48^ ^]^:

(2) $$i=k_{1}v+k_{2}v^{1/2}$$

Here, *k*_1_ *v* and *k*_2_ *v* ^1/2^ represent the degrees of control by the pseudocapacitance and ion diffusion, respectively.^[^ ^49^ ^]^ Figure 5d,e show that the capacitance contribution rates of Cu_2−x_Se‐PVP and Cu_2‐x_Se@N‐C at 0.6 mV s^−1^ are 79.2% and 88.7%, respectively. As the scan rate gradually increases from 0.1 to 0.6 mV s^−1^, the capacitance contribution rate of Cu_2−x_Se@N‐C increases from 78.3% to 88.7%, indicating that its electrochemical behaviors at high rates are mainly controlled by the capacitive current (Figure 5f). These fitting results confirm that the surface N‐doped carbon coating can endow Cu_2−x_Se@N‐C with a lower carrier impedance and rapid reaction kinetics, which is favorable in realizing high‐rate performance. To further ascertain the significance of the strong Se─C interactions between the N‐doped carbon layer and copper selenide, density functional theory calculations were conducted using the C layer, and each intermediate crystal phase involved in charging and discharging (details of the optimized model are shown in Figures S30–S33, Supporting Information). Figure 5g shows that the introduction of N‐doped carbon significantly reduces the binding energy of Cu‐Se within Cu_2−x_Se, indicating that the Cu_2−x_Se@N‐C structure displays excellent thermodynamic stability. The binding energies of copper selenide after carbon doping in various charge/discharge states reveal that Cu_2_Se@N‐C exhibits a stronger energy trend toward evolution into CuSe@N‐C, confirming the origin of the rapid electrode reaction kinetics (Figure 5h). Furthermore, the differential charge density of Cu_2−x_Se@N‐C reveals significant charge transfer from the N─C layer to Cu_2–x_Se (Figure 5i), with an interaction energy of −3.35 eV. This strong interaction effectively mitigates the dissolution of Se species, resulting in excellent cyclic stability.^[^ ^50^ ^]^ Compared to similar types of chalcogenide cathode materials, Cu_2−x_Se@N‐C exhibits significant advantages in terms of rate capability and cycling stability. Thus, the problems of sluggish reaction kinetics and continuous capacity fading associated with bulk S‐, Se‐, and Cu‐based chalcogenide compounds are effectively addressed (Table S1, Supporting Information).

## 3 Conclusion

In summary, we fabricated an ultrathin N‐doped carbon‐wrapped flower‐shaped Cu_2−x_Se@N‐C composite via simple γ radiation‐pyrolysis. The unique 3D morphology and amorphous C layer endowed copper selenide with a high efficiency and reversible Cu storage capacity. As a cathode material for aqueous Cu‐ion battery, Cu_2−x_Se@N‐C exhibited brilliant cyclic stability and rate performance. It could operate for >30 000 cycles at 5 A g^−1^ over 2000 h, with a negligible capacity decay rate of only 0.0002% per cycle. Additionally, Cu_2−x_Se@N‐C even displayed a high reversible capacity of 310.6 mAh g^−1^ at 20 A g^−1^. Ex situ XPS and XRD revealed the mechanisms of energy storage based on the multi‐electron redox reactions of the Cu and Se dual active sites of the Cu_2−x_Se@N‐C cathode and its mechanism of capacity decay. The strong interaction between the N‐doped carbon layer and Cu_2−x_Se via Se─C could effectively mitigate the volume expansion and persistent oxidative dissolution of Se during charging‐discharging. Additionally, the N‐doped carbon layer improved the electron/ion transport behavior close to the particle–electrolyte interface, accelerating the electrode reaction kinetics. This study provides an effective strategy for fabricating aqueous Cu‐Se batteries with extended cycle stabilities and deepens the understanding of the structural evolution of the electrodes within M‐Se‐based batteries.

## Conflict of Interest

The authors declare no conflict of interest.

## Supporting information



Supporting Information

## Data Availability

The data that support the findings of this study are available from the corresponding author upon reasonable request.

## References

[1] a) D. Bin , F. Wang , A. G. Tamirat , L. Suo , Y. Wang , C. Wang , Y. Xia , Adv. Energy. Mater. 2018, 8, 1703008;

[2] a) H. Shuai , R. Liu , W. Li , X. Yang , H. Lu , Y. Gao , J. Xu , K. Huang , Adv. Energy. Mater. 2023, 13, 2202992;

[3] W. Li , K. Wang , K. Jiang , Adv. Sci. 2020, 7, 2000761. 10.1002/advs.202000761 PMC7709974 33304742

[4] J. Zhang , Q. Lei , Z. Ren , X. Zhu , J. Li , Z. Li , S. Liu , Y. Ding , Z. Jiang , J. Li , Y. Huang , X. Li , X. Zhou , Y. Wang , D. Zhu , M. Zeng , L. Fu , ACS Nano. 2021, 15, 17748. 34714615 10.1021/acsnano.1c05725

[5] W. Wu , S. Wang , L. Lin , H.‐Y. Shi , X. Sun , Energy Environ. Sci. 2023, 16, 4326.

[6] X. Lin , J. Zhang , H. Yan , J. Xu , Z. Miao , G. Shu , S. Zhao , T. Zhang , H. Yu , L. Yan , L. Zhang , J. Shu , Proc. Natl. Acad. Sci. 2023, 120, e2312091120. 37812706 10.1073/pnas.2312091120 PMC10589612

[7] Z. Miao , J. Xu , C. Xu , J. Zhang , Y. Liu , B. Wanyan , H. Yu , L. Yan , L. Zhang , J. Shu , Proc. Natl. Acad. Sci. 2023, 120, e2307646120. 37579150 10.1073/pnas.2307646120 PMC10450428

[8] W. Li , D. Wang , Adv. Mater. 2023, 35, 2304983. 10.1002/adma.202304983 37467467

[9] a) X. Wang , L. Liu , Z. Hu , C. Peng , C. Han , W. Li , Adv. Energy. Mater. 2023, 13, 2302927;

[10] X. Li , J. Liang , J. T. Kim , J. Fu , H. Duan , N. Chen , R. Li , S. Zhao , J. Wang , H. Huang , X. Sun , Adv. Mater. 2022, 34, 2200856. 10.1002/adma.202200856 35365923

[11] Z. Chen , F. Mo , T. Wang , Q. Yang , Z. Huang , D. Wang , G. Liang , A. Chen , Q. Li , Y. Guo , X. Li , J. Fan , C. Zhi , Energy. Environ. Sci. 2021, 14, 2441.

[12] J. Zhang , X. Zhang , C. Xu , Y. Liu , J. Xu , Z. Miao , H. Yu , L. Yan , L. Zhang , J. Shu , Proc. Natl. Acad. Sci. 2023, 120, e2220792120. 36940321 10.1073/pnas.2220792120 PMC10068761

[13] C. Dai , L. Hu , H. Chen , X. Jin , Y. Han , Y. Wang , X. Li , X. Zhang , L. Song , M. Xu , H. Cheng , Y. Zhao , Z. Zhang , F. Liu , L. Qu , Nat. Commun. 2022, 13, 1863. 35387998 10.1038/s41467-022-29537-5 PMC8987094

[14] a) J. Zhang , X. Zhang , C. Xu , H. Yan , Y. Liu , J. Xu , H. Yu , L. Zhang , J. Shu , Adv. Energy. Mater. 2022, 12, 2103998;

[15] J. Li , Y. Ren , Z. Li , Y. Huang , ACS Nano. 2023, 17, 18507. 37710357 10.1021/acsnano.3c06361

[16] Y. Yang , J. Xiao , J. Cai , G. Wang , W. Du , Y. Zhang , X. Lu , C. C. Li , Adv. Funct. Mater. 2021, 31, 2005092.

[17] Y. Wang , B. Wang , J. Zhang , D. Chao , J. Ni , L. Li , Carbon. Energy. 2023, 5, e261.

[18] Z. Qiao , Y. Xie , J. Xu , X.‐M. Liu , Y.‐J. Zhu , Y. Qian , Can. J. Chem. 2000, 78, 1143.

[19] X. Zhang , Y. Jin , K. Zhang , Q. Yuan , H. Wang , M. Jia , J. Colloid. Interface. Sci. 2023, 630, 786. 36356446 10.1016/j.jcis.2022.10.166

[20] M. Zhang , K. Huang , Y. Zou , J. Jia , L. Wu , W. Zeng , Chem. Eng. J. 2024, 499, 156547.

[21] S. Li , H. Duan , J. Yu , C. Qiu , R. Yu , Y. Chen , Y. Fang , X. Cai , S. Yang , ACS Catal. 2022, 12, 9074.

[22] Y. Tang , X. Wang , J. Chen , X. Wang , D. Wang , Z. Mao , Carbon. 2021, 174, 98.

[23] R. Yu , T. Ren , K. Sun , Z. Feng , G. Li , C. Li , J. Phys. Chem. C. 2009, 113, 10833.

[24] S. Sun , Z. Han , W. Liu , Q. Xia , L. Xue , X. Lei , T. Zhai , D. Su , H. Xia , Nat. Commun. 2023, 14, 6662. 37863930 10.1038/s41467-023-42335-x PMC10589268

[25] F. Monjezi , F. Jamali‐Sheini , R. Yousefi , J. Alloys. Compd. 2019, 780, 626.

[26] L. Yue , D. Wang , Z. Wu , W. Zhao , Y. Ren , L. Zhang , B. Zhong , N. Li , B. Tang , Q. Liu , Y. Luo , A. M. Asiri , X. Guo , X. Sun , Chem. Eng. J. 2022, 433, 134477.

[27] P. Zhou , F. Xiao , R. Weng , Q. Huang , L. Wang , Q. He , W. Tang , P. Yang , R. Su , P. He , B. Jia , L. Bian , J. Mater. Chem. A. 2022, 10, 10514.

[28] K. Zhu , S. Wei , Q. Zhou , S. Chen , Y. Lin , P. Zhang , Y. Cao , C. Wang , Y. Wang , Y. Xia , D. Cao , Z. Mohamed , X. Guo , X. Yang , X. Wu , L. Song , Nano Res. 2023, 16, 2421.

[29] a) Z.‐g. Xia , J.‐j. Zhang , M.‐q. Fan , C.‐j. Lv , Z. Chen , C. Li , New. Carbon. Mater. 2023, 38, 190;

[30] a) S. Xiao , Z. Li , J. Liu , Y. Song , T. Li , Y. Xiang , J. S. Chen , Q. Yan , Small. 2020, 16, 2002486; 10.1002/smll.202002486 32964603

[31] Q. Lv , W. Si , J. He , L. Sun , C. Zhang , N. Wang , Z. Yang , X. Li , X. Wang , W. Deng , Y. Long , C. Huang , Y. Li , Nat. Commun. 2018, 9, 3376. 30139938 10.1038/s41467-018-05878-y PMC6107639

[32] C. Zhang , H. Gu , Y. Hu , W. Zhang , Z. Li , Chem. Eng. J. 2023, 474, 145688.

[33] Z. Ye , Y. Jiang , T. Yang , L. Li , F. Wu , R. Chen , Adv. Sci. 2022, 9, 2103456. 10.1002/advs.202103456 PMC8728854 34708583

[34] S. Li , J. Yu , S. Zhang , W. Qiu , X. Tang , Z. Lin , R. Cai , Y. Fang , S. Yang , X. Cai , Adv. Funct. Mater. 2024, 34, 2311989.

[35] H. Hu , J. Wang , B. Cui , X. Zheng , J. Lin , Y. Deng , X. Han , Angew. Chem., Int. Ed. 2022, 61, e202114441. 10.1002/anie.202114441 34806271

[36] Y. Ma , S. Qing , H. Liu , C. Ma , Y. Yu , C. Yu , L. Wang , J. Energy. Chem. 2025, 100, 409.

[37] a) H. Jia , X. Li , J. Song , X. Zhang , L. Luo , Y. He , B. Li , Y. Cai , S. Hu , X. Xiao , C. Wang , K. M. Rosso , R. Yi , R. Patel , J.‐G. Zhang , Nat. Commun. 2020, 11, 1474; 32193387 10.1038/s41467-020-15217-9 PMC7081208

[38] C. Lu , L. Liu , S. He , B. Li , Z. Du , H. Du , X. Wang , S. Zhang , W. Ai , Adv. Energy. Mater. 2024, 14, 2401221.

[39] a) E. Filippo , D. Manno , A. Serra , J. Alloys. Compd. 2012, 538, 8;

[40] T. Wang , W. Zeng , J. Zhu , W. Tian , J. Wang , J. Tian , D. Yuan , S. Zhang , S. Mu , Nano Energy. 2023, 113, 108577.

[41] a) Z. Hao , X. Shi , Z. Yang , L. Li , S.‐L. Chou , Adv. Funct. Mater. 2022, 32, 2208093;

[42] L. Liu , B. Li , J. Wang , H. Du , Z. Du , W. Ai , Small. 2024, 20, 2309647. 10.1002/smll.202309647 38240559

[43] a) H. Peng , Y. Zhang , Y. Chen , J. Zhang , H. Jiang , X. Chen , Z. Zhang , Y. Zeng , B. Sa , Q. Wei , J. Lin , H. Guo , Mater. Today. Energy. 2020, 18, 100519;

[44] K. Tang , X. Yu , J. Sun , H. Li , X. Huang , Electrochim. Acta. 2011, 56, 4869.

[45] a) S. Wang , S. Jiao , J. Wang , H.‐S. Chen , D. Tian , H. Lei , D.‐N. Fang , ACS Nano. 2017, 11, 469; 27977919 10.1021/acsnano.6b06446

[46] a) D. Yu , X. Wei , D. Zhao , S. Gao , G. Zhao , H. Zhang , Z. Li , M. Yu , Y. Sun , Electrochim. Acta. 2022, 404, 139703;

[47] S. Li , C. Huang , L. Gao , Q. Shen , P. Li , X. Qu , L. Jiao , Y. Liu , Angew. Chem., Int. Ed. 2022, 61, e202211478. 10.1002/anie.202211478 36260436

[48] Y. Cao , Y. Zhu , C. Du , X. Yang , T. Xia , X. Ma , C. Cao , ACS Nano. 2022, 16, 1578. 35023721 10.1021/acsnano.1c10253

[49] Y. Sun , Z. Lian , Z. Ren , Z. Yao , Y. Yin , P. Huai , F. Zhu , Y. Huang , W. Wen , X. Li , R. Tai , D. Zhu , ACS Nano. 2021, 15, 14766. 34432437 10.1021/acsnano.1c04636

[50] Z. Zhou , X. Hu , Y. Liu , S. Li , W. Guan , Z. Du , W. Ai , ACS Appl. Mater. Interfaces. 2024, 16, 4530. 38241522 10.1021/acsami.3c12755

