# Supplementary material for: Tailoring the Structural Evolution of Multi‐Electron Redox Conversions via Strong Selenium–Carbon Interaction for Robust Aqueous Copper‐Ion Batteries
Source: Adv Sci (Weinh). 2025 Feb 20;12(15):2417084. doi: 10.1002/advs.202417084 (PMC12005742; doi:10.1002/advs.202417084)
Supplement: Supplementary file 1 — Supporting Information [file ADVS-12-2417084-s001.docx]

Supporting Information

**Tailoring the Structural Evolution of Multi-Electron Conversion Redox Conversions via Strong Selenium–Carbon Interaction for Robust Aqueous Copper-Ion Batteries.**

Fan Jiang, Haoyu Peng, Yiqian Wu, Yichen Li, Zeyu Zhang, Yue Wang, Jiuqiang Li, Jing Peng, Maolin Zhai*

1. **Experimental Section**

**Synthesis of Cu_2–x_Se and Cu_2–x_Se-PVP nanoflowers**^[1]^All reagents were utilized directly without further purification. Initially, 0.19 g copper acetate (Aladdin) was dissolved in 12 mL ammonia solution (7.5 vol%) (Beijing Tong Guang Fine Chemical Co. Ltd.) to form a blue copper-ammonia complex. This was followed by the addition of 2 mL 0.25 M sodium selenosulfate solution, 5 mL isopropanol (Tianjing Concord Technology Co. Ltd.), 250 mg polyvinylpyrrolidone (PVP, Mreda), and deionized water, ensuring the total volume reached 35 mL. The solution was bubbled with nitrogen for 15 minutes to exclude air, after which the solution was sealed and subjected to γ-ray irradiation with ^60^Co source (Department of Applied Chemistry, Peking University). The total absorbed dose was maintained at 50 kGy (the dose rate verified using Fricke dosimeter). After irradiation, the sample was collected via filtration, washed by ultrapure water, 10% acetic acid solution and anhydrous ethanol several times, and finally dried under vacuum at 60 °C for 12 hours. Cu_2–x_Se was prepared using the same methods as described above, without the addition of PVP.

**Synthesis of Cu_2–x_Se@N-C** Cu_2–x_Se-PVP powder was placed into porcelain boat at middle of tube furnace. Firstly, the sample underwent calcination at 280 °C for 1h under argon atmosphere with heating rate of 5 °C min^-1^ and then the temperature was elevated to 500 °C for another two hours. The nitrogen-doped carbon encapsulated Cu_2_**_–_**_x_Se@N-C-500 (abbreviated Cu_2_**_–_**_x_Se@N-C) was collected after cooling naturally to room temperature. For comparison, Cu_2_**_–_**_x_Se@N-C-400 and Cu_2_**_–_**_x_Se@N-C-600 samples were pyrolyzed at 400 °C and 600 °C for 2 h, respectively.

**Material characterization** The composition of the samples was characterized by X-ray diffraction (XRD, X-Pert3 Powder) with Cu-Kα radiation (λ=1.54178 Å), and Raman spectroscopy (DXRxi) with 532 nm laser. The microstructure and morphology of the samples were investigated by field emission scanning electron microscopy (FSEM, Hitachi S-4800) and high-resolution transmission electron microscopy (HRTEM, Tecnai 2100F) along with energy-dispersive X-ray spectroscopy (EDS) for image acquisition. The chemical states of the samples were analyzed by X-ray photoelectron spectroscopy (XPS, AXIS Supra) with an Al-Kα excitation source and charge correction performed with the C 1s peak at 284.8 eV. The specific surface area, porosity, and pore size distribution of the materials were measured using ASAP2010 through nitrogen adsorption-desorption isotherms at constant temperature. Quantitative elemental analysis of the samples was conducted by thermogravimetric analysis (TG, Q600 SDT) under air atmosphere in range of 20-800 °C with heating rate of 10 °C min^-1^, and by inductively coupled plasma atomic emission spectroscopy (ICP-AES, Prodigy 7). The fine electronic structure and coordination structure were further analyzed through X-ray absorption spectroscopy, with the XANES and the EXAFS processed using Athena software. The signal of lattice cation vacancies was captured through electron paramagnetic resonance (EPR, Bruker EMXplus-6/1).

**Electrochemical measurements** The sample powder was mixed uniformly with Super P and PVDF (dissolved in N-methyl pyrrolidone (NMP)) in a mass ratio of 8:1:1, followed by the addition of NMP as solvent and stirring for 6 hours. The black slurry was then coated onto titanium foil and dried at 60 °C under vacuum for 12 hours as working electrode with mass loading of 1.5-2 mg cm^-2^. The sample was used as the cathode, copper foil as the anode, and glass fiber membrane (Whatman, GF/D) as the separator, with a 0.5 M CuSO_4_ solution as the electrolyte, to assemble the type R2032 coin cell. Galvanostatic charge-discharge test and Galvanostatic Intermittent Titration Technique (GITT) test were both conducted on the Land CT3002A cell test system with the voltage range between 0-0.4 V vs. Cu^2+^/Cu. During GITT test, charging/discharging at 0.5 A g^-1^ for 2 minutes was followed by a 2-minutes relaxation to reach ion diffusion equilibrium. The diffusion coefficient of copper ions *D*_Cu_^2+^ was calculated by Equation S1:

$$D_{Cu^{2+}}=\frac{4}{\pi\tau}\left(\frac{n_{m}V_{m}}{S}\right)^{2}\left[\frac{\Delta E_{S}}{\Delta E_{t}}\right]^{2}$$

*τ* represents relaxation time, *n_m_* and *V_m_* respectively denote the molar amount and molar volume of the active material, *S* is the geometric area electrode, Δ*E_s_* is the total voltage change after relaxation, and Δ*E_t_* is the voltage change during charge-discharge process.

Cyclic voltammetry (CV) tests and Electrochemical Impedance Spectroscopy (EIS, frequency range from 100 kHz to 0.01 Hz, AC amplitude of 10 mV) were performed on an electrochemical workstation (AUTOLAB PGSTAT302). CV tests at different scan rates (0.1 mV s^-1^, 0.2 mV s^-1^, 0.4 mV s^-1^, 0.6 mV s^-1^) were employed to analyze the charge-discharge polarization behavior and the storage mechanism. All electrochemical tests above were controlled at room temperature (25 °C).

**Ex situ experiments** After charging/discharging the battery to different voltages, the cathode was disassembled. They were washed with deionized water and ethanol, and then dried at 60 °C under vacuum. Samples from different charging/discharging stages were utilized directly for ex situ XPS, ex situ XRD and ex situ Raman tests. The sample powders were scraped off from the titanium foil, dispersed in ethanol, and drop-cast onto carbon grid for ex situ TEM test.

**Computational Details** All the calculations are performed in the framework of the density functional theory with the projector augmented plane-wave method, as implemented in the Vienna ab initio simulation package^[2]^. The generalized gradient approximation proposed by Perdew-Burke-Ernzerhof (PBE) is selected for the exchange-correlation potential^[3]^. The cut-off energy for plane wave is set to 480 eV. The energy criterion is set to 10^−4^ eV in the iterative solution of the Kohn-Sham equation. All the structures are relaxed until the residual forces on the atoms have declined to less than 0.05 eV/Å. To avoid interlaminar interactions, a vacuum spacing of 20 Å is applied perpendicular to the slab. The binding energy *E*_bind_ is expressed as Equation S2：

$$\Delta E_{bind}=E_{A+B}-E_{A}-E_{B}$$

where *E_A+B_* is the total energy of slab A model with B molecule, *E_A_* is the energy of a A slab, and *E_B_* is that for a B molecule.

Here, we define $\Delta\rho\text{=}\rho_{\text{A+B}}-\rho_{\text{A}}-\rho_{\text{B}}$ as the charge density difference of A/B heterostructure, where *ρ*_A/B_, *ρ*_A_ and *ρ*_B_ are the charge densities of A/B heterostructure, isolated A and B slabs, respectively.  Constructing a N-doped carbon model that incorporates the three different types of nitrogen atoms (pyridinic N, pyrrolic N, and graphitic N) in a graphene. The models of heterostructures used in this work were based on the (110) of Cu_2_Se crystal (space group: Fm-3m. a=b=c=5.802 Å, a=β=γ=90°), (102) of CuSe crystal (space group: P63/mmc. a=b=3.658 Å, c=15.106 Å, a=β=90°, γ=120°), (110) of Cu_1.8_Se crystal (space group: Fm-3m. a=b=c=5.746 Å, a=β=γ=90°), and (1 0 0) of Cu_3_Se_2_ crystal (space group: F-421m. a=b=6.406 Å, c=4.279 Å, a=β=γ=90°)


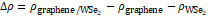

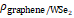

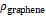

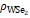


1. **Supplementary Figures**


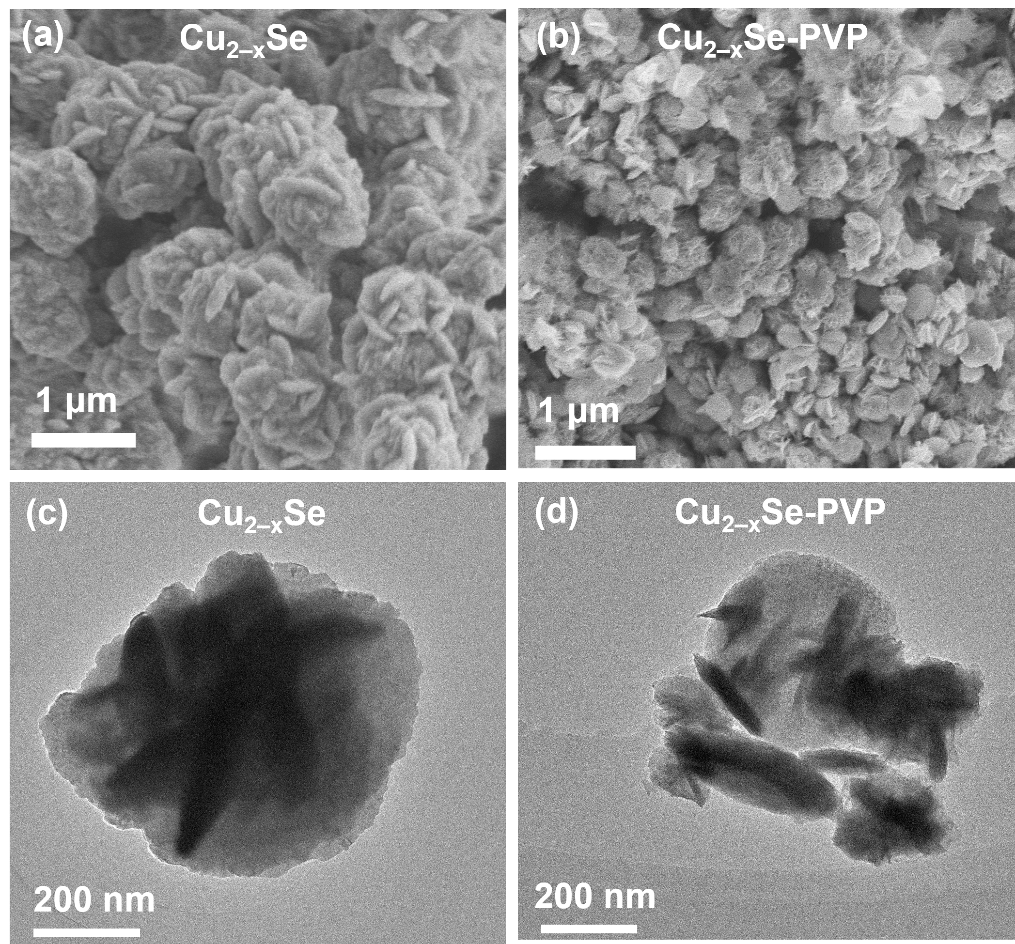


**Figure S1.** a-b) SEM images of Cu_2–x_Se and Cu_2–x_Se-PVP and c-d) TEM images of Cu_2–x_Se and Cu_2–x_Se-PVP.


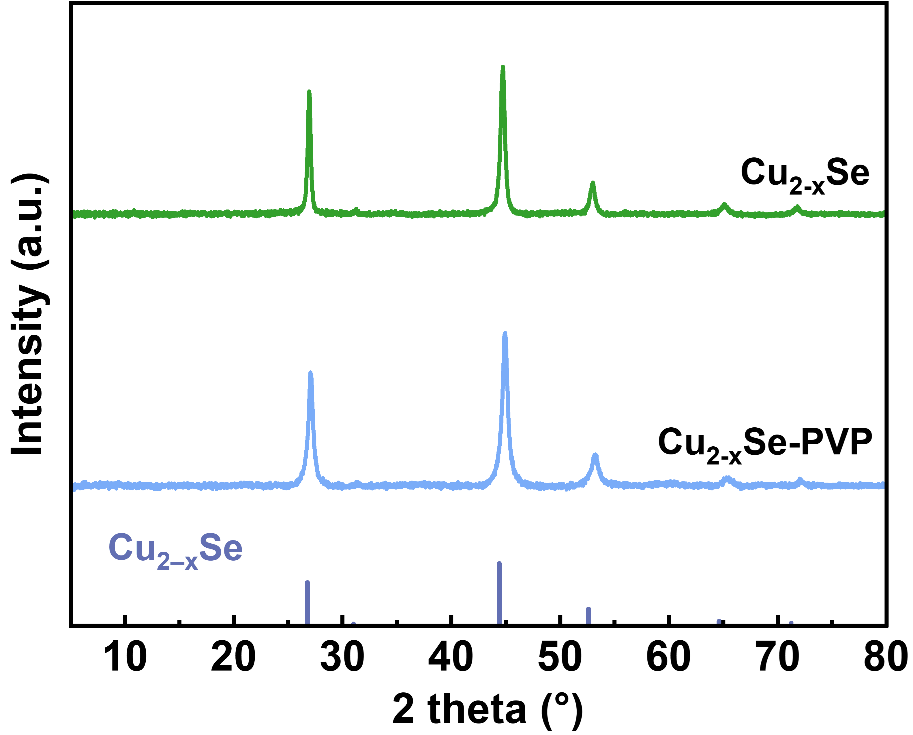


**Figure S2**. XRD patterns of Cu_2–x_Se and Cu_2–x_Se-PVP.


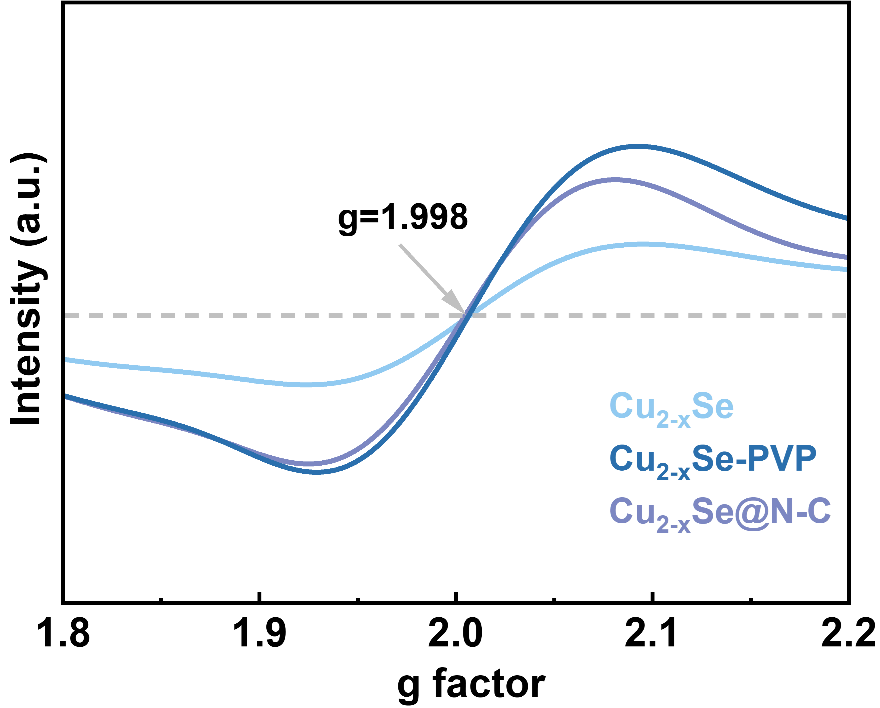


**Figure S3.** EPR plots of Cu_2–x_Se, Cu_2–x_Se-PVP and Cu_2–x_Se@N-C.


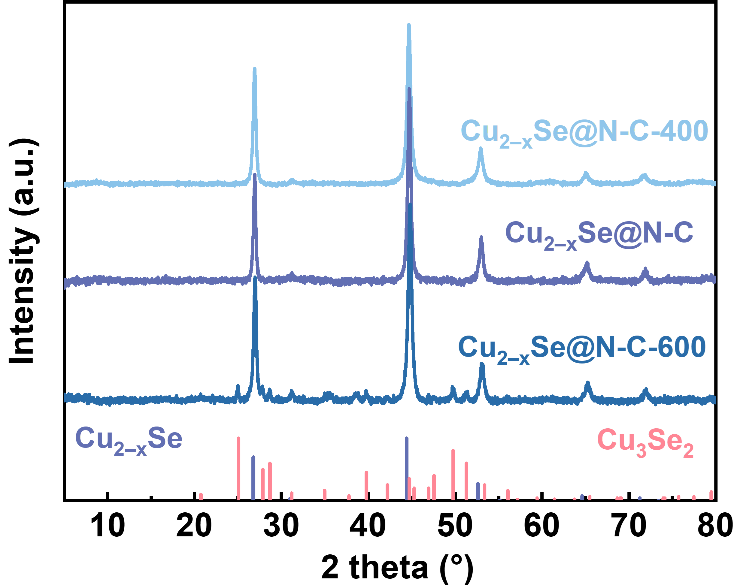


**Figure S4.** XRD patterns of Cu_2–x_Se@N-C-400, Cu_2–x_Se@N-C and Cu_2–x_Se@N-C-600.


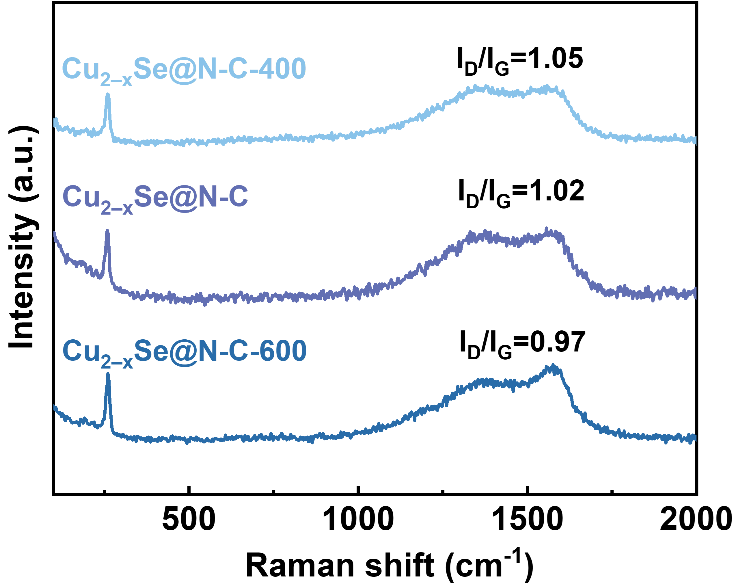


**Figure S5.** Raman spectrum of Cu_2–x_Se@N-C-400, Cu_2–x_Se@N-C and Cu_2–x_Se@N-C-600.


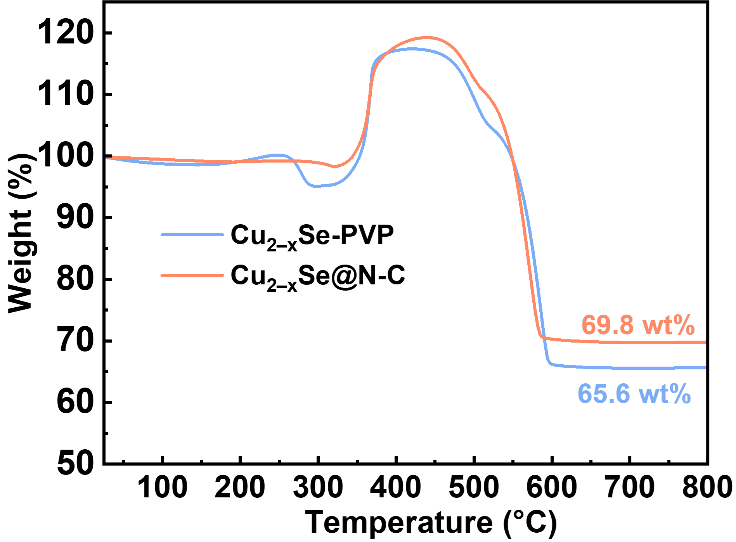


**Figure S6.** Thermogravimetric curves of Cu_2–x_Se-PVP and Cu_2–x_Se@N-C.


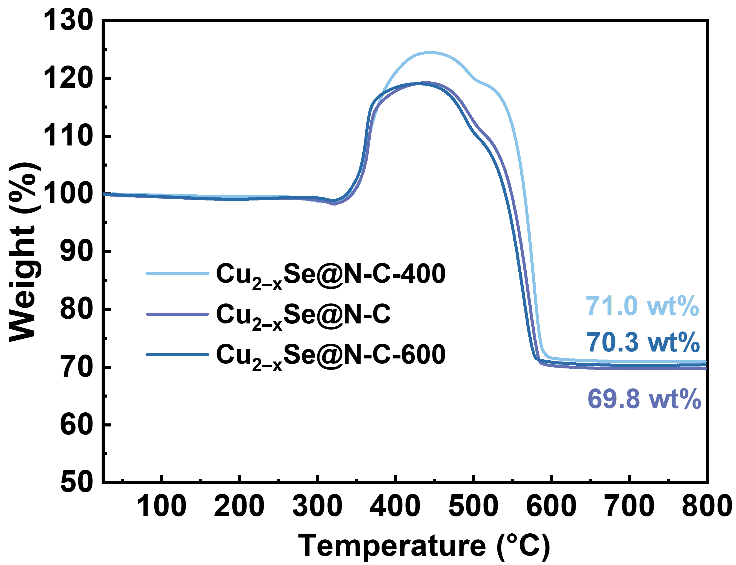


**Figure S7.** TG profiles of Cu_2–x_Se@N-C-400, Cu_2–x_Se@N-C and Cu_2–x_Se@N-C-600.


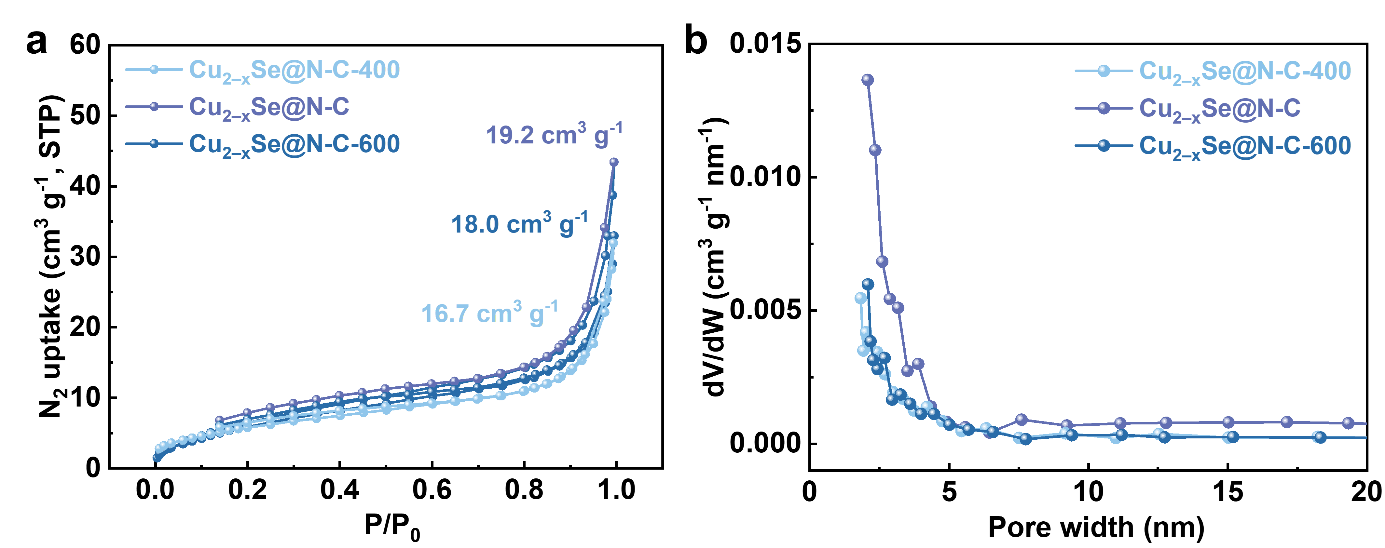


**Figure S8.** a) Nitrogen adsorption–desorption isotherms and b) the corresponding pore size distribution plots of Cu_2–x_Se@N-C-400, Cu_2–x_Se@N-C and Cu_2–x_Se@N-C-600.


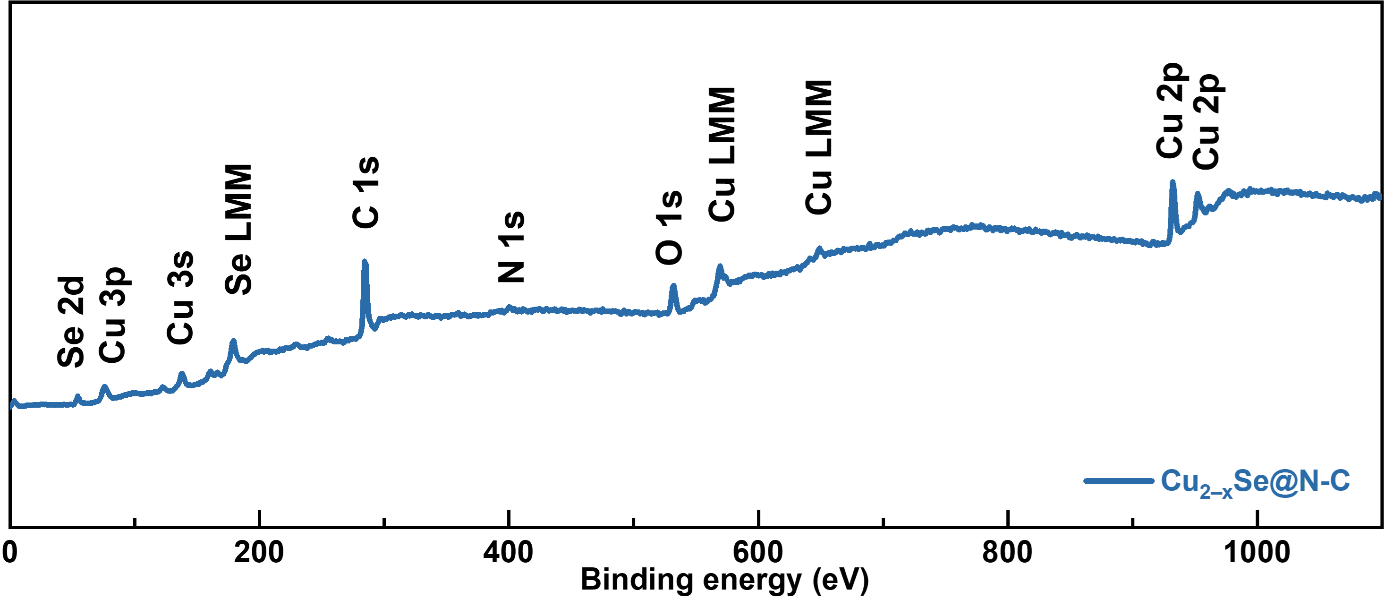


**Figure S9.** Survey XPS spectrums of Cu_2–x_Se@N-C.


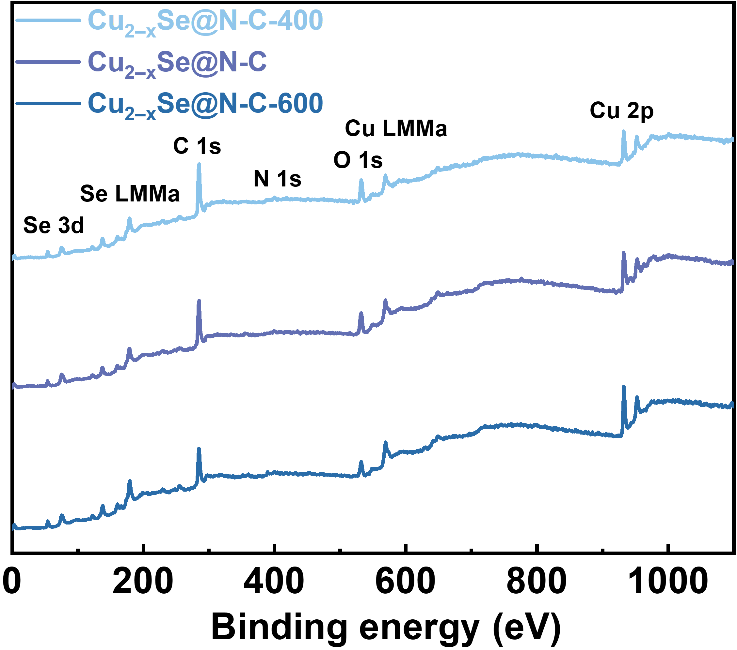


**Figure S10.** Survey XPS spectrums of Cu_2–x_Se@N-C-400, Cu_2–x_Se@N-C and Cu_2–x_Se@N-C-600.


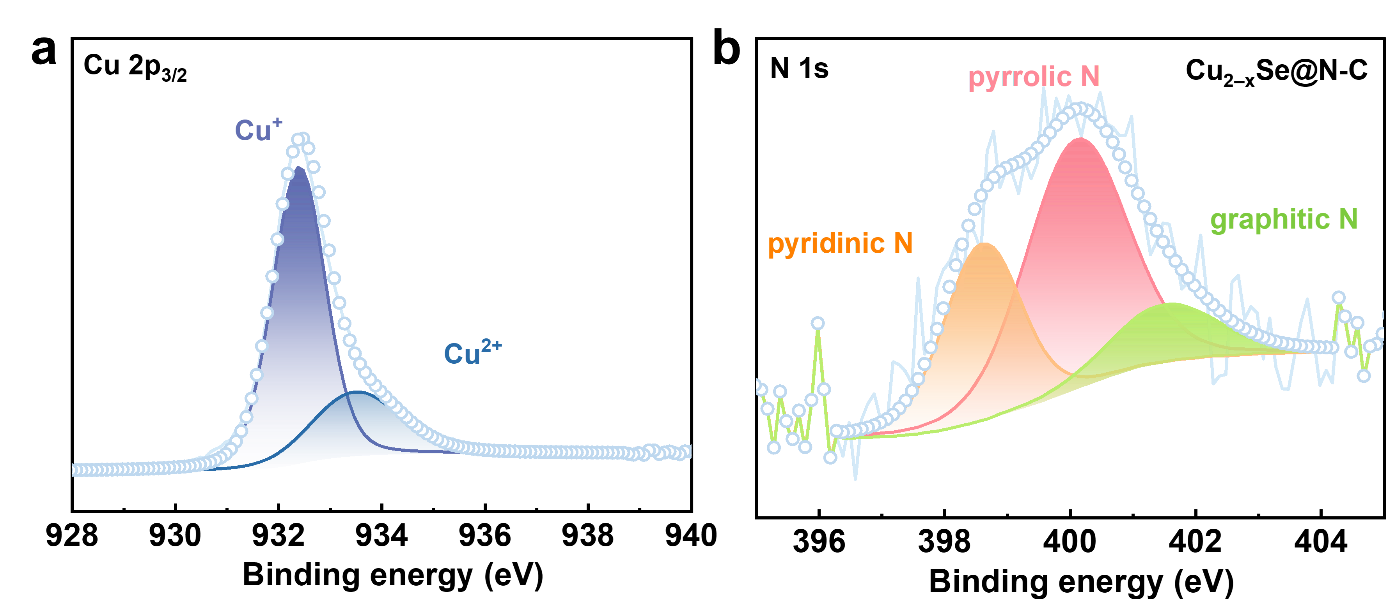


**Figure S11.** a) Cu 2p_3/2_ and b) N 1s spectrums of Cu_2–x_Se@N-C.


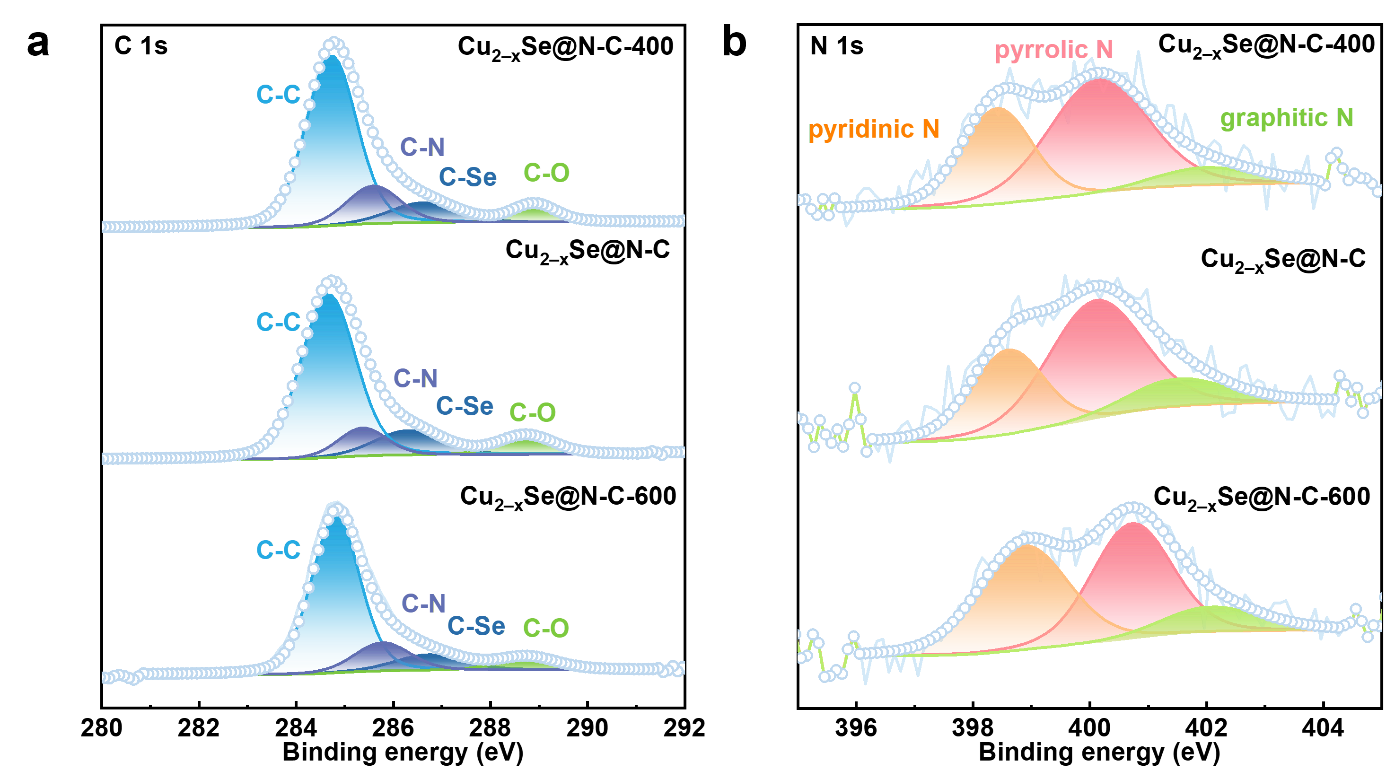


**Figure S12.** a) The high-resolution C 1s spectrums and b) N 1s of Cu_2–x_Se@N-C-400, Cu_2–x_Se@N-C and Cu_2–x_Se@N-C-600.


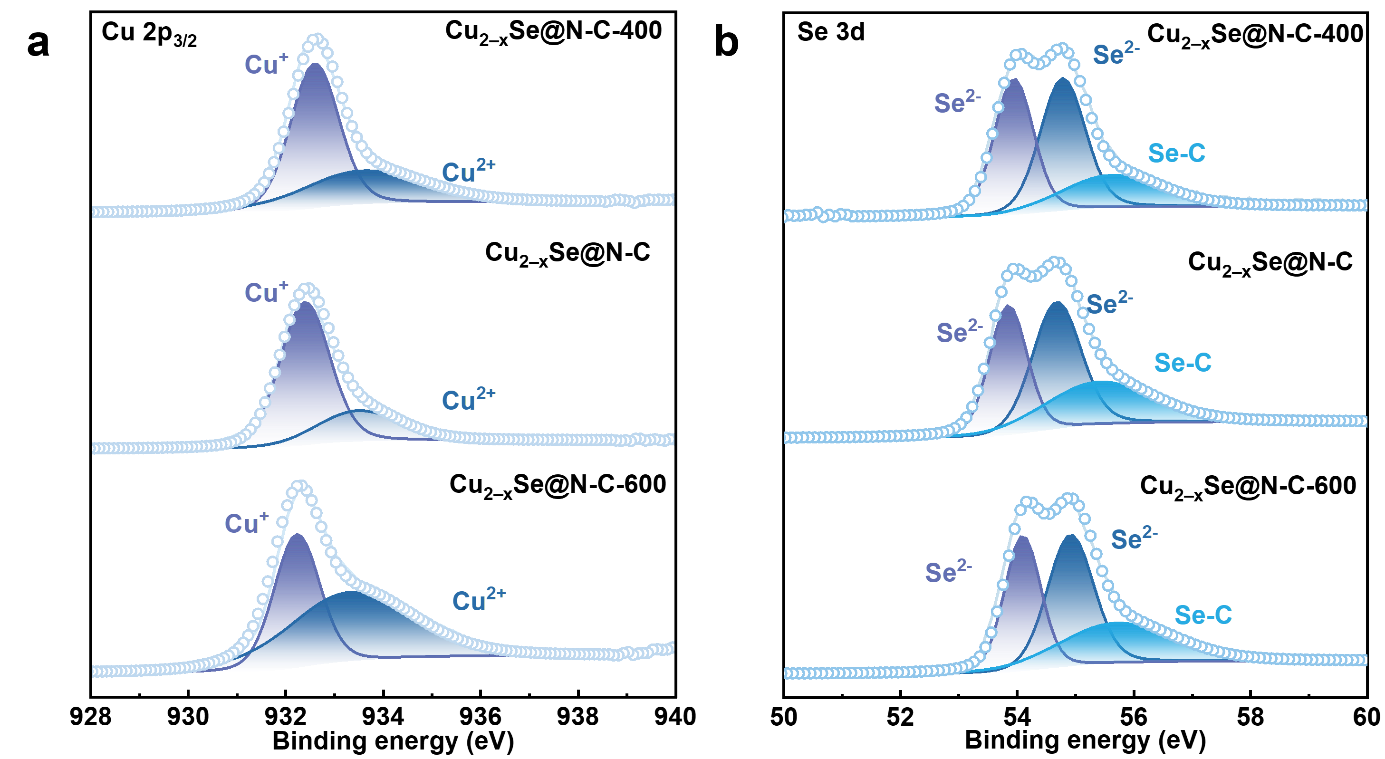


**Figure S13.** a) The high-resolution Cu 2p_3/2_ spectrums and b) Se 3d of Cu_2–x_Se@N-C-400, Cu_2–x_Se@N-C and Cu_2–x_Se@N-C-600.


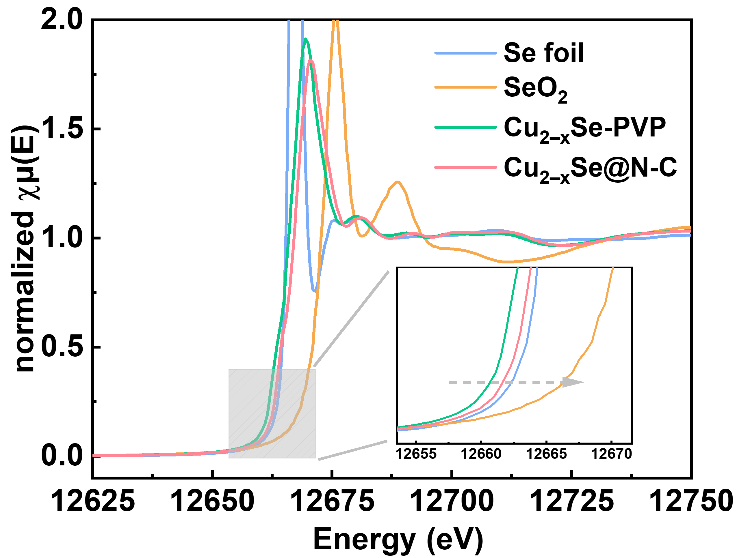


**Figure S14.** Se K-edge XANES spectra of Cu_2–x_Se-PVP and Cu_2–x_Se@N-C.


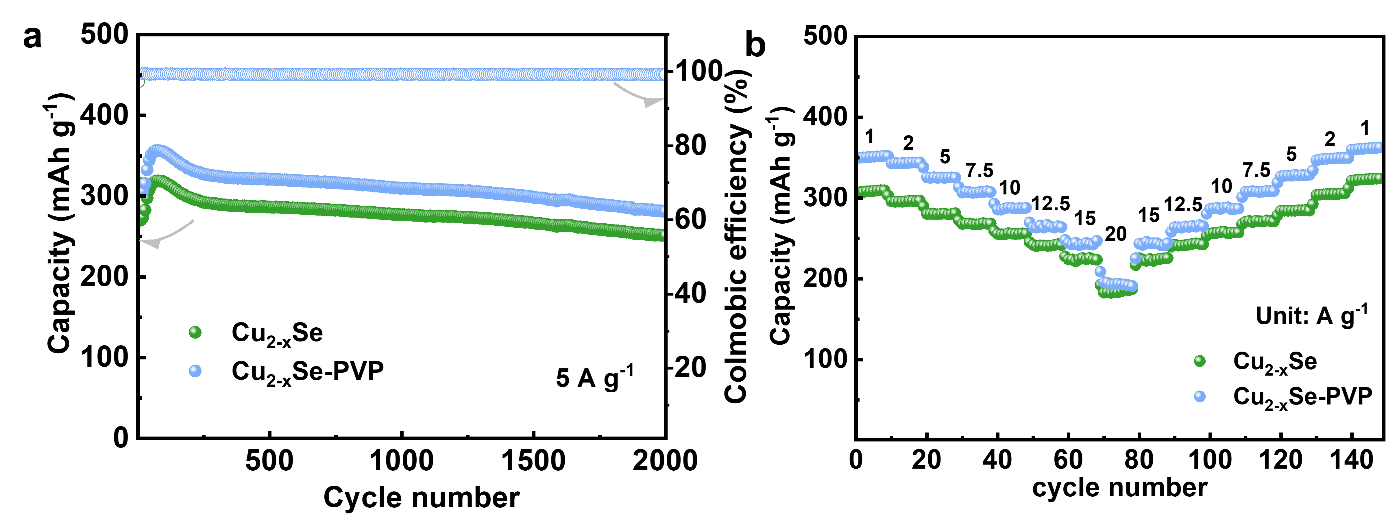


**Figure S15.** a) Cycling performance at 5.0 A g^-1^ and b) rate performance of Cu_2–x_Se and Cu_2–x_Se-PVP.


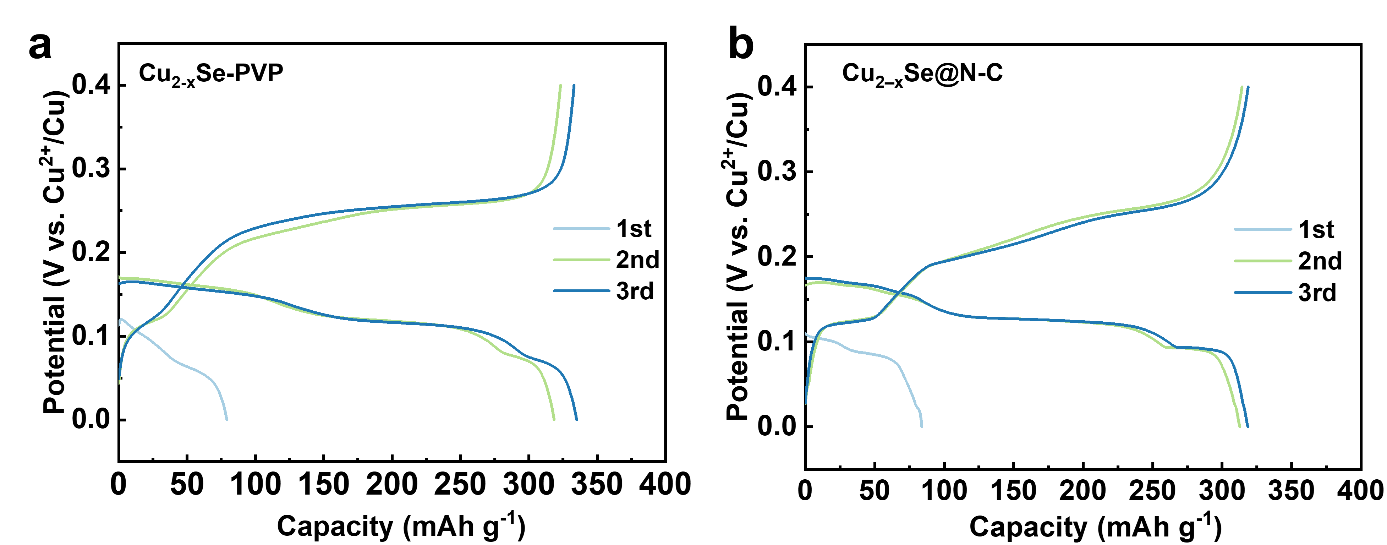


**Figure S16.** GCD profiles of a) Cu_2–x_Se-PVP and b) Cu_2–x_Se@N-C at 1^st^-3^rd^ cycle.


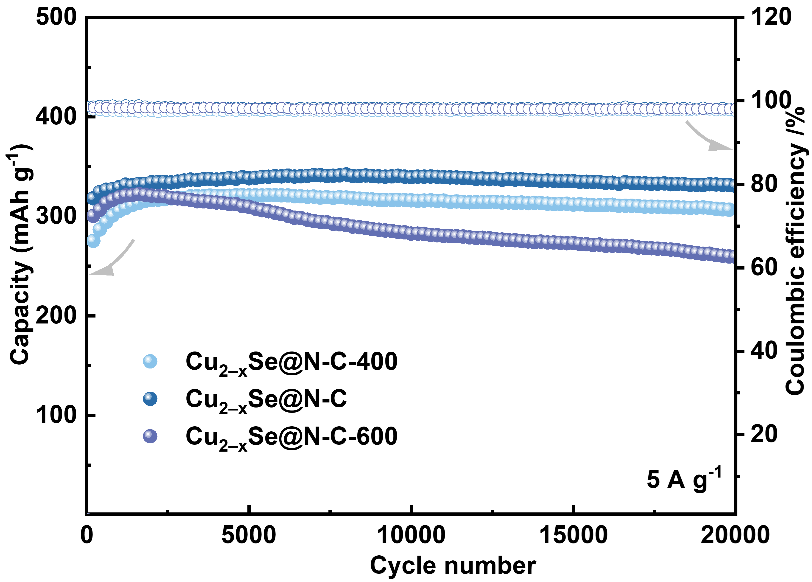


**Figure S17.** Cycling performance of Cu_2–x_Se@N-C-400, Cu_2–x_Se@N-C and Cu_2–x_Se@N-C-600 at 5.0 A g^-1^.


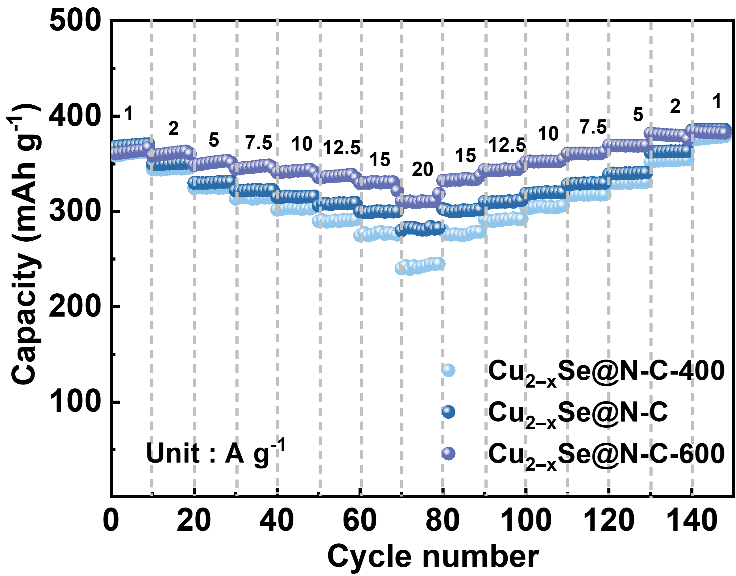


**Figure S18.** Rate performance of Cu_2–x_Se@N-C-400, Cu_2–x_Se@N-C and Cu_2–x_Se@N-C-600.


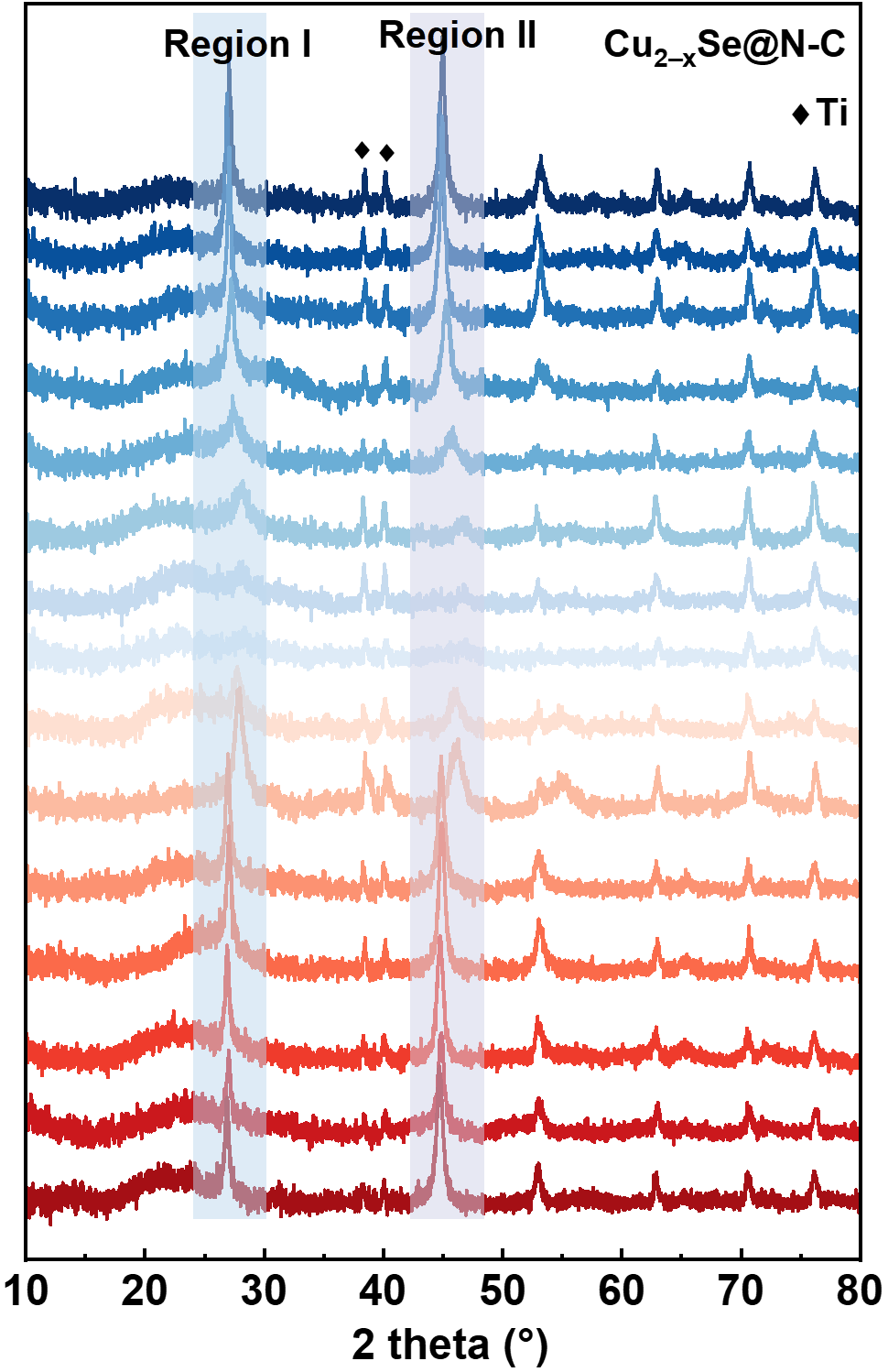


**Figure S19.** Ex-situ XRD pattern of Cu_2–x_Se@N-C during 1^st^ cycle and 2^nd^ discharge process.


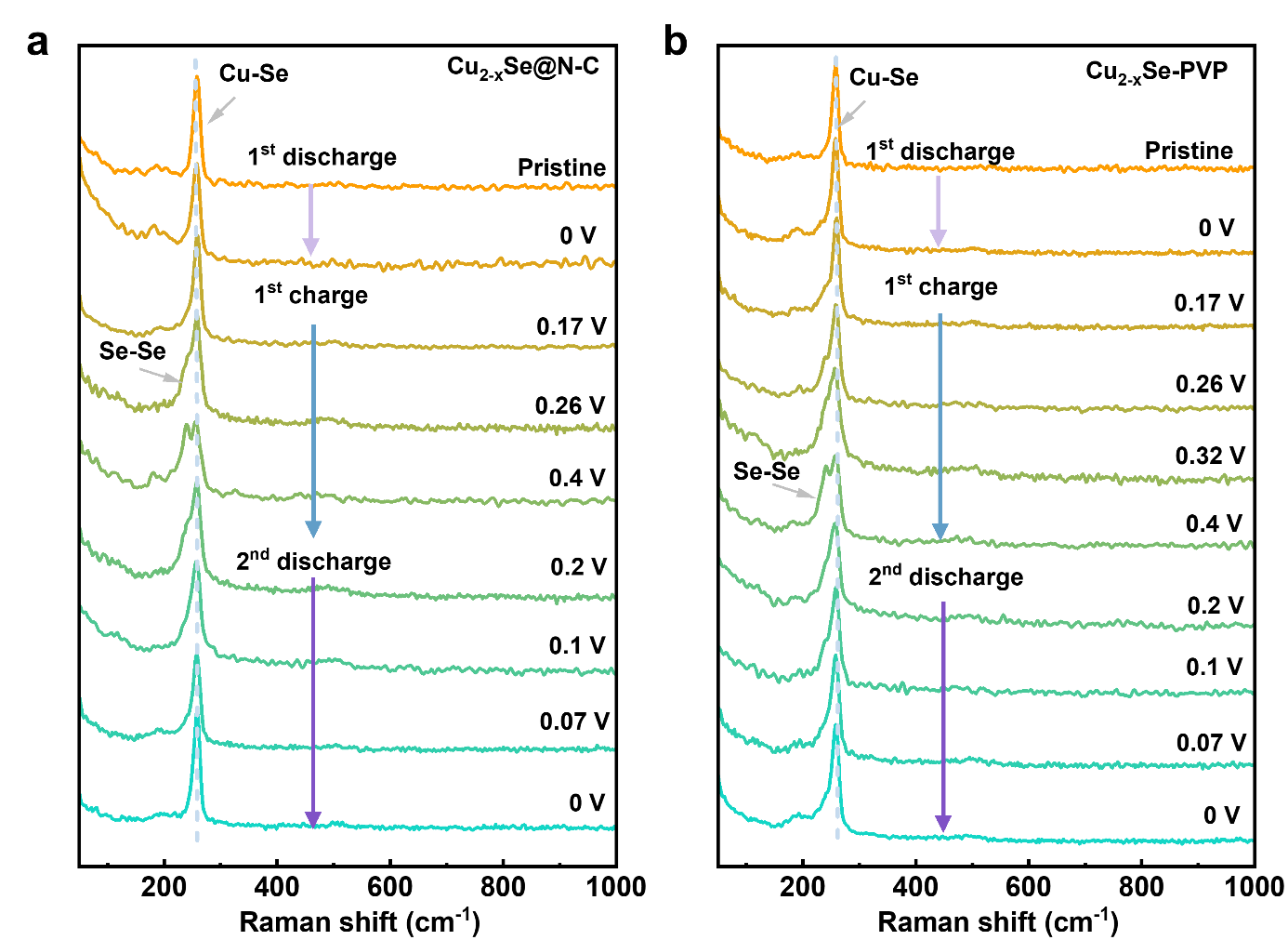


**Figure S20** Ex-situ Raman spectrums of a) Cu_2-x_Se@N-C and b) Cu_2–x_Se-PVP during 1^st^ cycle and 2^nd^ discharge process.


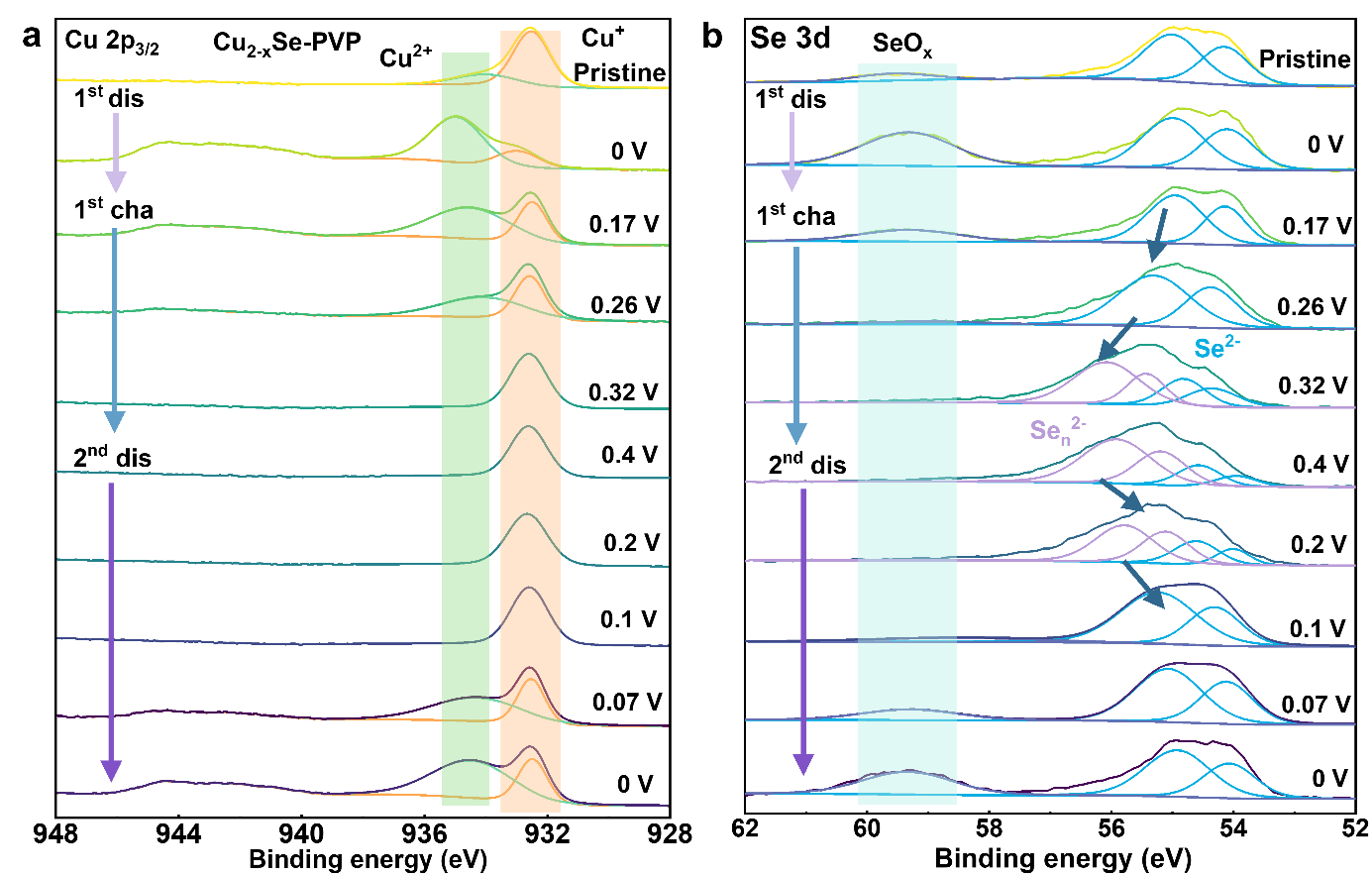


**Figure S21.** Ex situ a) Cu 2p_3/2_ and b) Se 3d XPS spectra of the Cu_2–x_Se-PVP cathode during the 1^st^ cycle and 2^nd^ discharge.


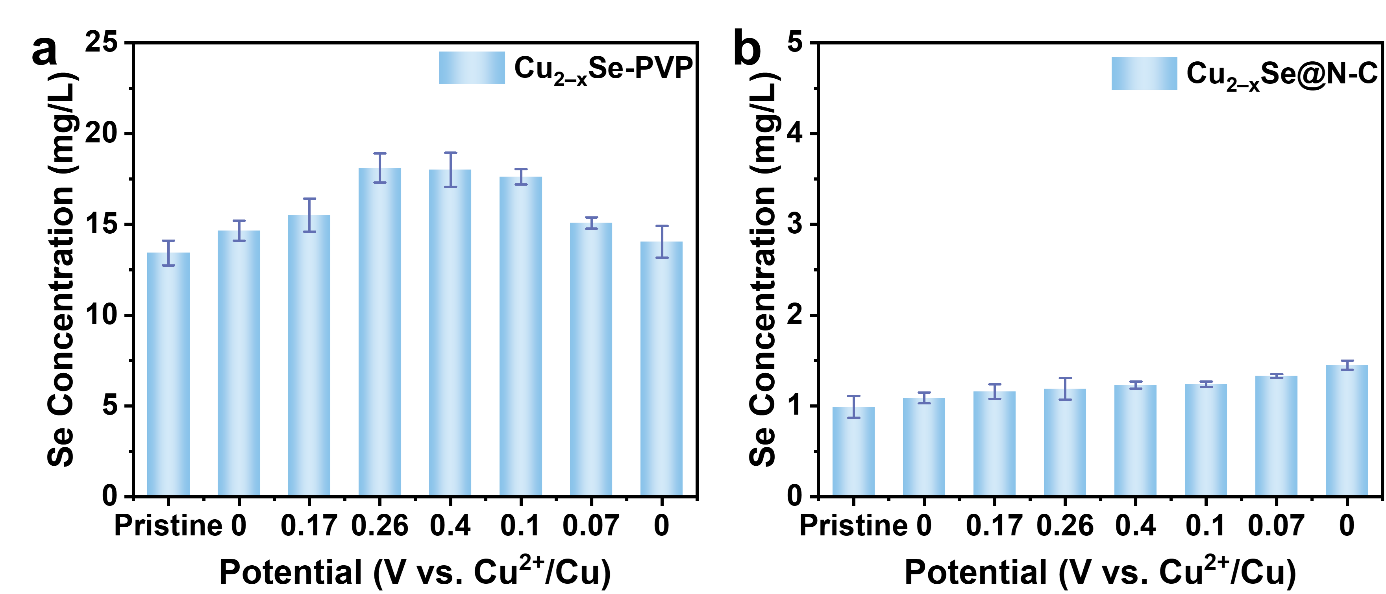


**Figure S22.** The content of Se element in 0.5 M CuSO_4_ for the various discharge/charge states of a) Cu_2–x_Se-PVP and b) Cu_2–x_Se@N-C cathode.


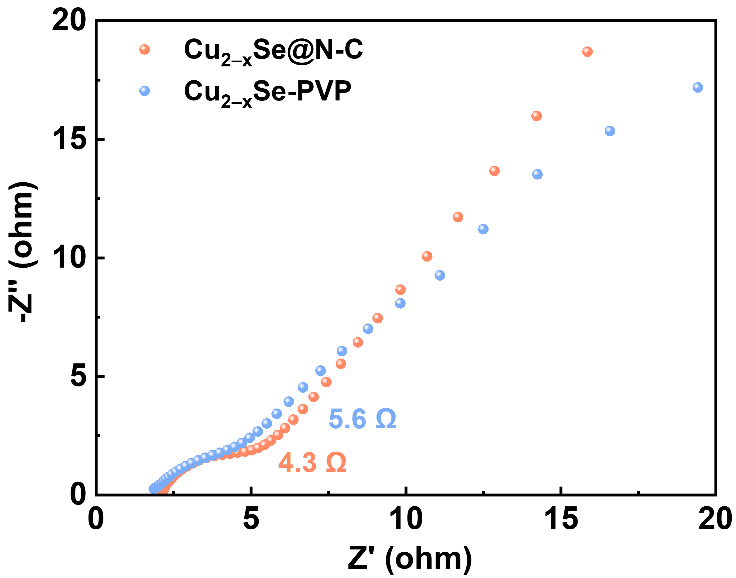


**Figure S23.** EIS profiles of Cu_2–x_Se-PVP and Cu_2–x_Se@N-C.


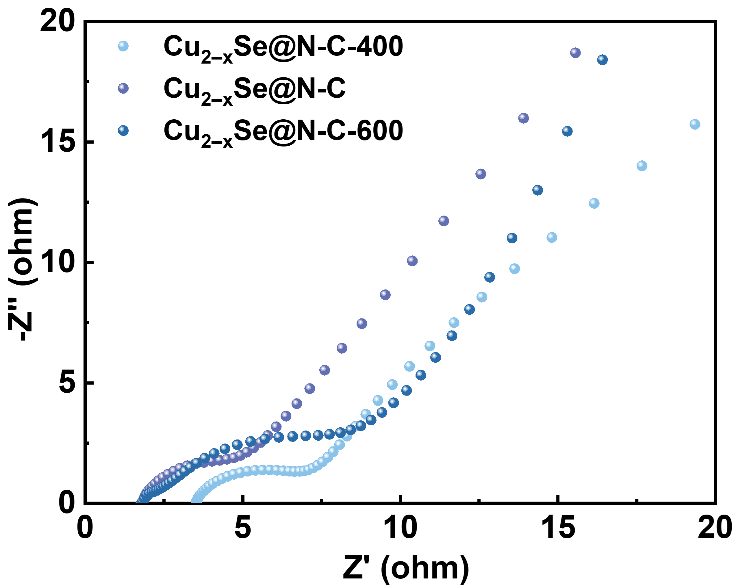


**Figure S24.** EIS spectrums of Cu_2–x_Se@N-C-400, Cu_2–x_Se@N-C and Cu_2–x_Se@N-C-600.


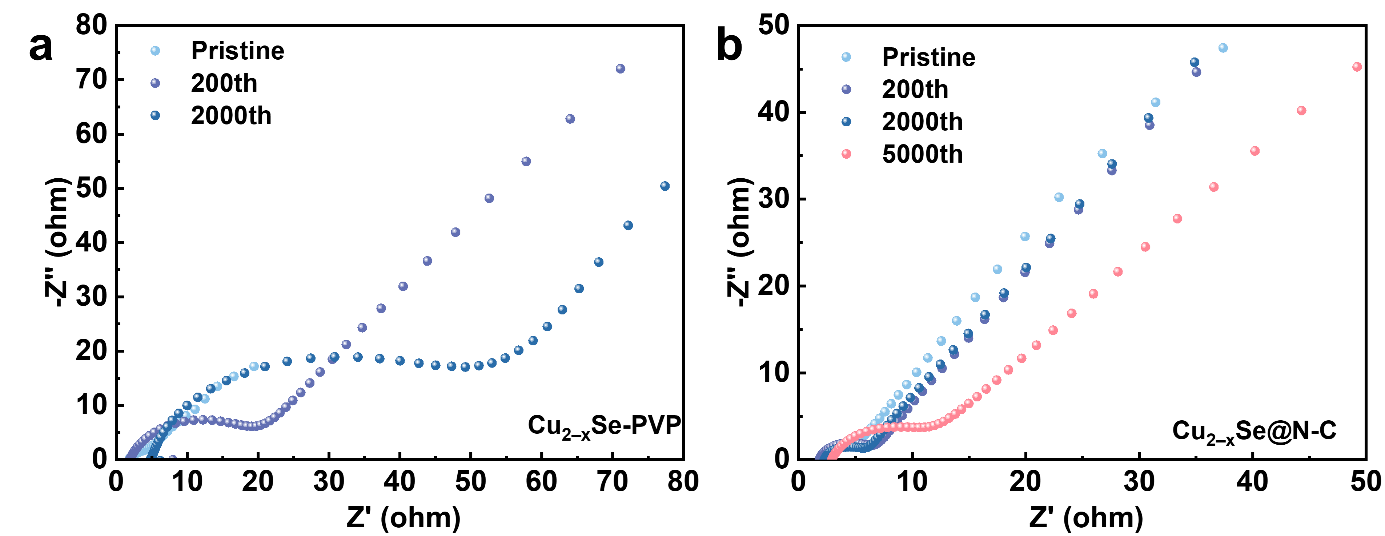


**Figure S25.** EIS plots of the a) Cu_2–x_Se-PVP and b) Cu_2–x_Se@N-C electrodes at various cycling number.


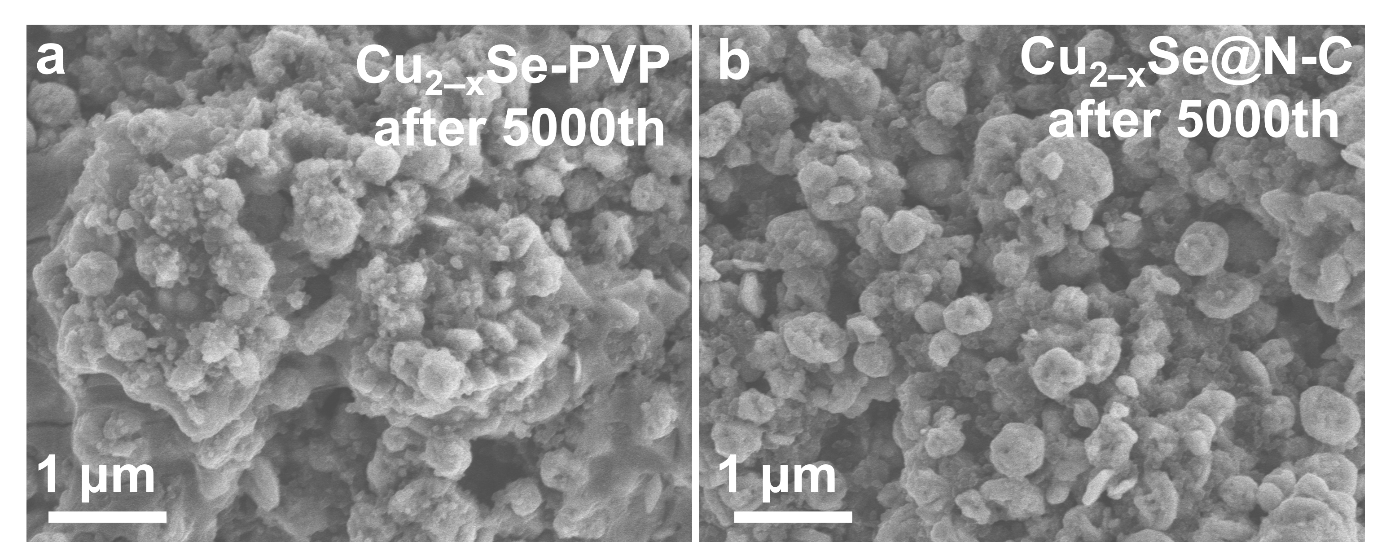


**Figure S26.** SEM images of a) Cu_2–x_Se-PVP and b) Cu_2–x_Se@N-C cathode after 5000^th^ cycle.


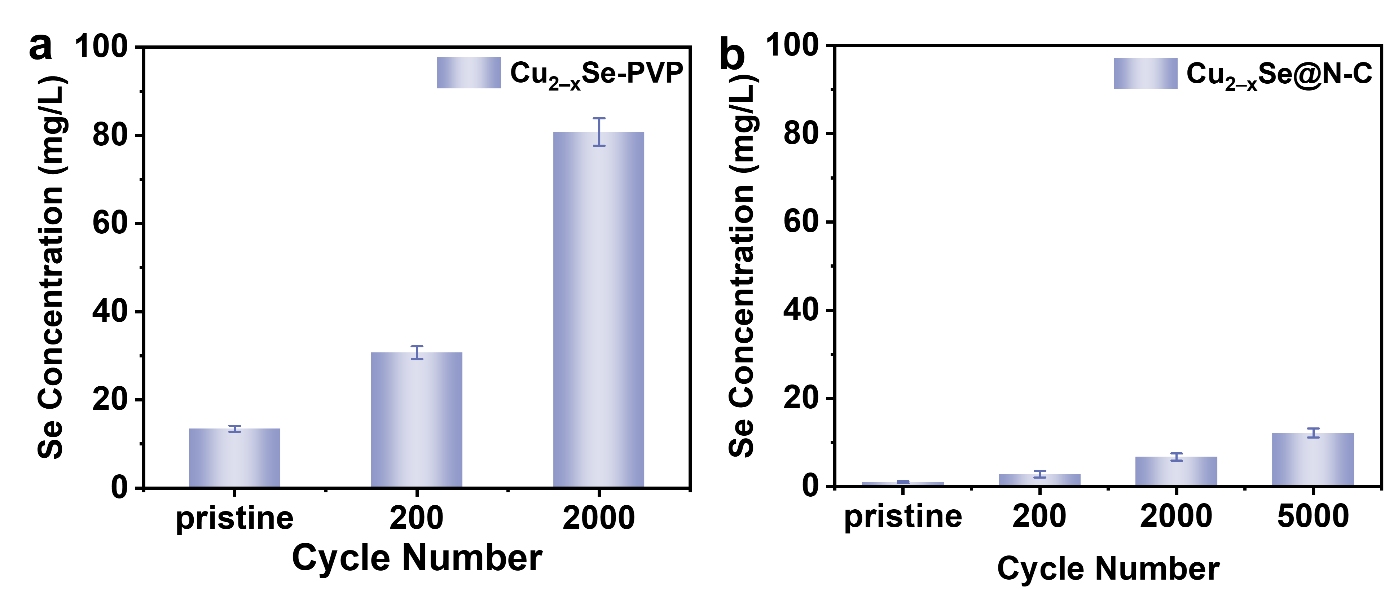


**Figure S27.** The content of Se element in 0.5 M CuSO_4_ for the various cycles of a) Cu_2–x_Se-PVP and b) Cu_2–x_Se@N-C cathode.


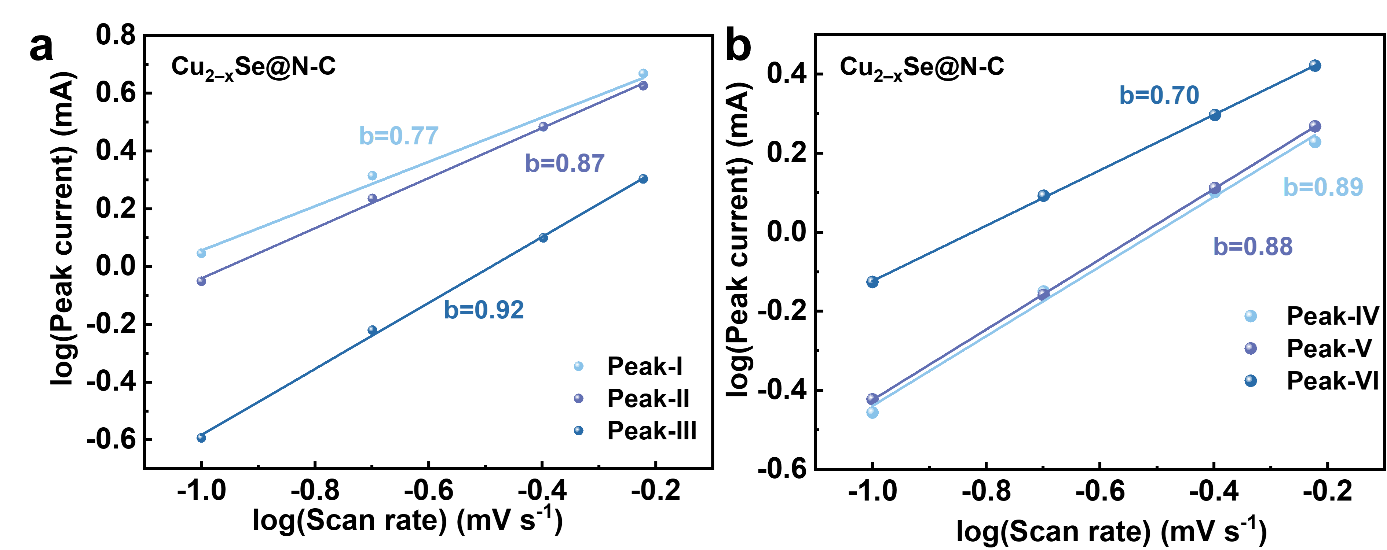


**Figure S28.** Log(*i*) versus log(*v*) plots of Cu_2–x_Se@N-C based on a) Peak-I, Peak-II, Peak-III and b) Peak-IV, Peak-V, Peak-VI.


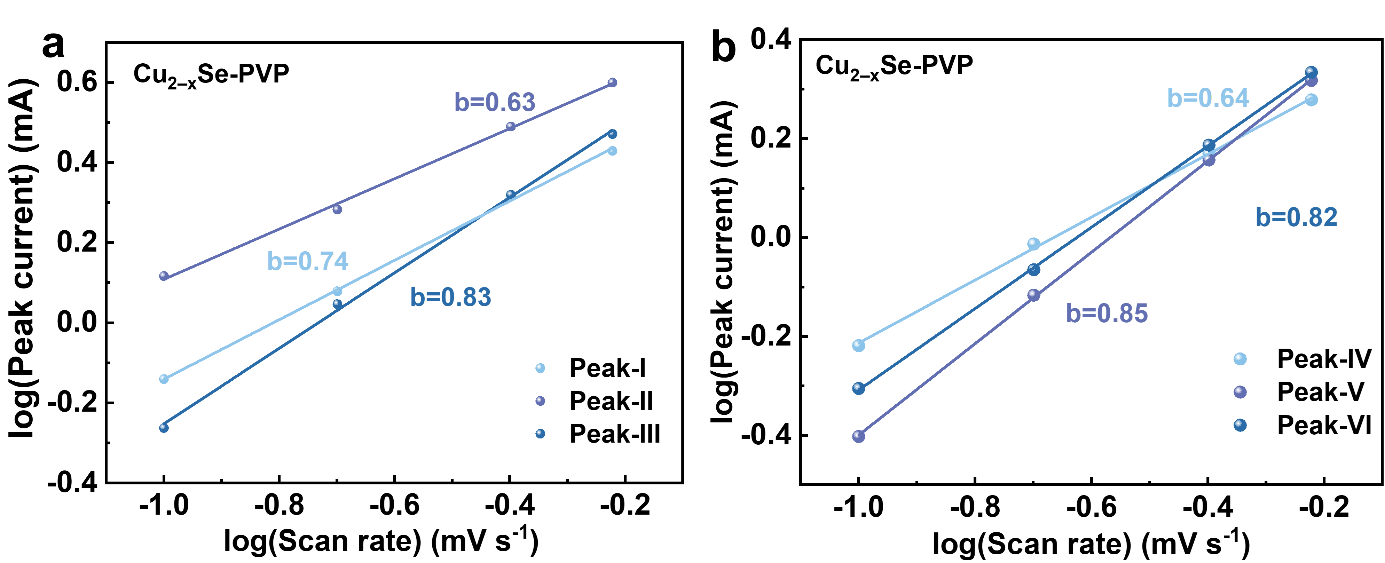


**Figure S29.** Log(*i*) versus log(*v*) plots of Cu_2–x_Se-PVP based on a) Peak-I, Peak-II, Peak-III and b) Peak-IV, Peak-V, Peak-VI.

**
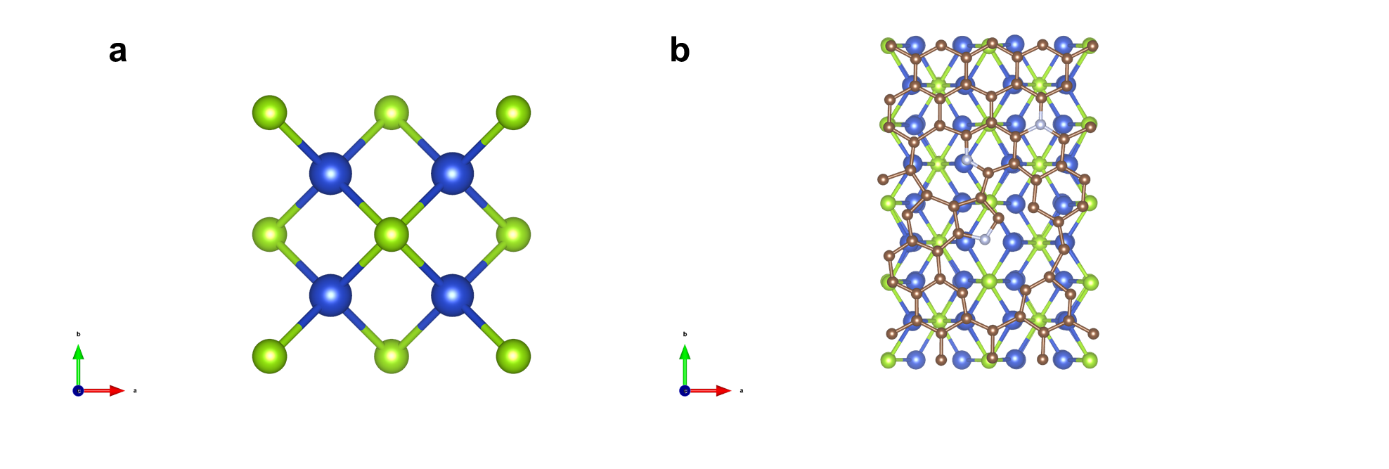
**

**Figure S30.** Top view of optimization model of a) Cu_2_Se and b) Cu_2_Se@N-C.

**
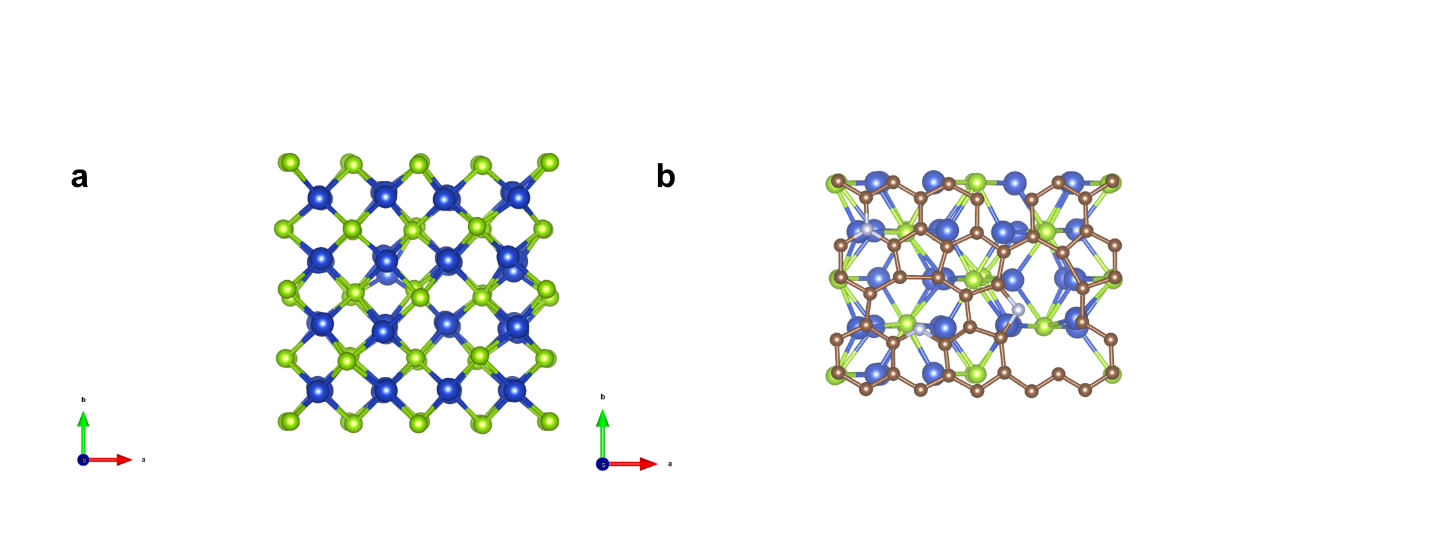
**

**Figure S31.** Top view of optimization model of a) Cu_1.8_Se and b) Cu_1.8_Se@N-C.

**
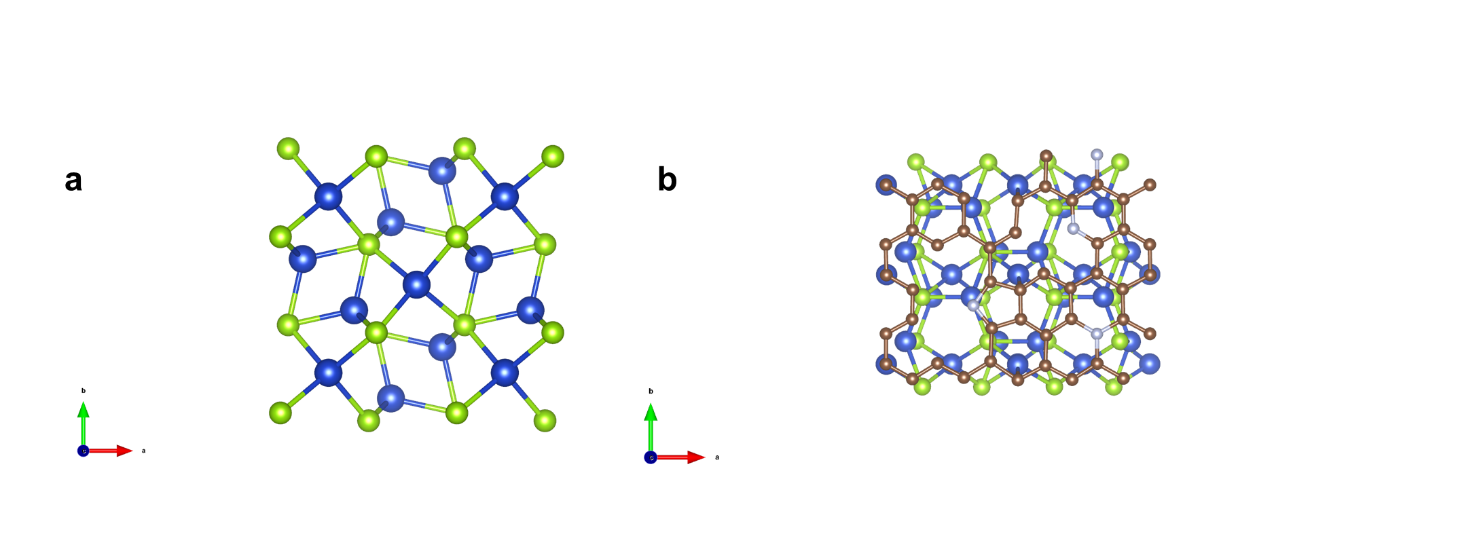
**

**Figure S32.** Top view of optimization model of a) Cu_3_Se_2_ and b) Cu_3_Se_2_@N-C.

**
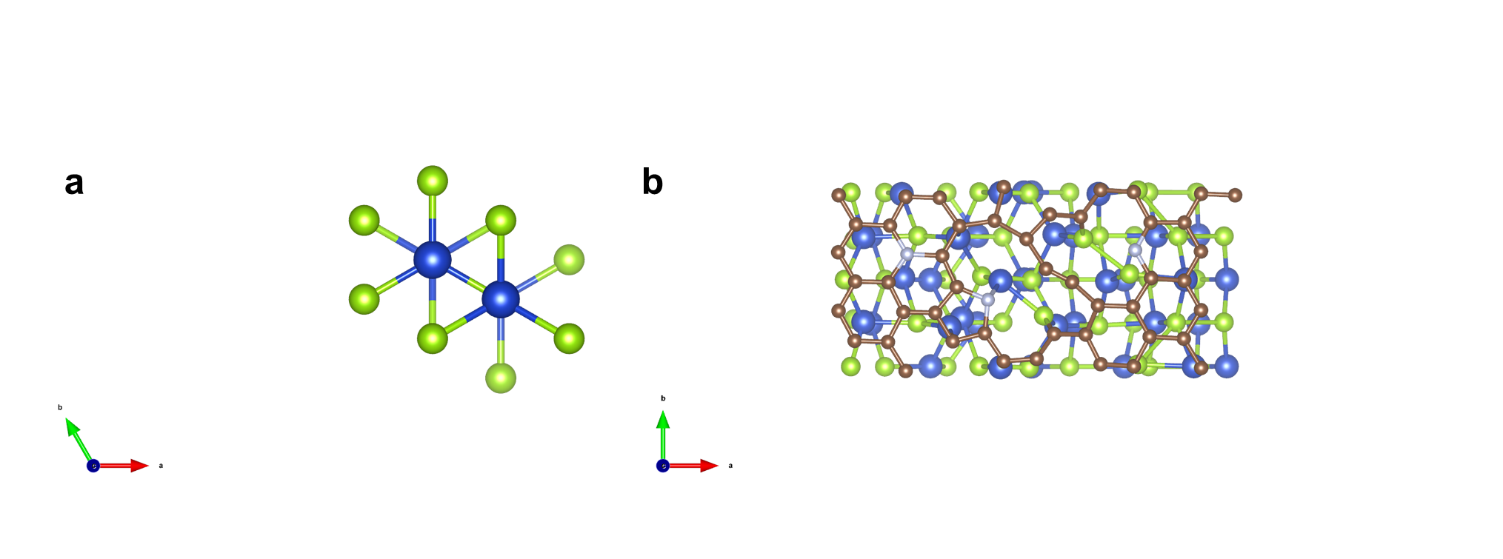
**

**Figure S33.** Top view of optimization model of a) CuSe and b) CuSe@N-C.

**Table S1.** Performance comparison of with other aqueous Cu-chalcogen batteries and other M-Se batteries (M=Zn, Al, Ca).

| **Sample** | **Battery**  **Type** | **Capacity/Rate**  **(mAh g^-1^/A g^-1^)** | **Retention/Cycles**  **(Rate)** | **Refs.** |
| --- | --- | --- | --- | --- |
| Cu_2_**_–_**_x_Se@N-C | Cu-Se | 310.6 / 20  (base on CuSe) | ~92.9%/30000  (5 A g^-1^) | This Work |
| Se/CMK-3^[a]^ | Cu-Se | 310.0 / 4  (base on CuSe) | 81%/400  (4 A g^-1^) | ^[4]^ |
| Se@NCP-30^[b]^ | Cu-Se | 314.0 / 2  (base on CuSe) | 89.7%/1500  (2 A g^-1^) | ^[5]^ |
| Se@C-48 | Cu-Se | 305 / 10  (base on CuSe) | 95%/300  (2 A g^-1^) | ^[6]^ |
| CuSe | Cu-Se | 285 / 20 | 90%/30000  (5 A g^-1^) | ^[7]^ |
| CuS | Cu-S | 463 / 5 | 91%/2500  (5 A g^-1^) | ^[8]^ |
| CuS_0.5_Se_0.5_ | Cu-S | 395 / 20 | >90%/>1000  (10 A g^-1^) | ^[9]^ |
| Te | Cu-Te | 628.3 / 0.5 | 80.4%/2000  (20 A g^-1^) | ^[10]^ |
| Ru doped  Se | Zn-Se | 71 / 10 | 88.1%/800  (5 A g^-1^) | ^[11]^ |
| Cu_2_**_–_**_x_Se | Zn-Se | 150 / 0.5 | 91%>/20000  (5 A g^-1^) | ^[12]^ |
| Se NWs@CC^[c]^ | Al-Se | 260 / 0.05 | 93%/100  (0.1 A g^-1^) | ^[13]^ |
| Se/CMK-3 | Ca-Se | >350 / 0.3 | ~54%/50  (0.3 A g^-1^) | ^[14]^ |

^[a]^ CMK-3: ordered mesoporous carbon ; ^[b]^ NCP-30: nanoporous carbon powder ; ^[c]^ NWs: nanowires; CC: carbon cloth.

References

[1] S. Singh, M. C. Rath, A. K. Singh, T. Mukherjee, O. D. Jayakumar, A. K. Tyagi, S. K. Sarkar, *Radiat. Phys. Chem.* **2011**, *80*, 736-741.

[2] G. Kresse, D. Joubert, *Physical Review B* **1999**, *59*, 1758-1775.

[3] J. P. Perdew, K. Burke, M. Ernzerhof, *Phys. Rev. Lett.* **1996**, *77*, 3865-3868.

[4] Z. Wu, X. Lin, J. Zhang, X. Chu, J. Xu, J. Li, Y. Liu, H. Yu, L. Yan, L. Zhang, J. Shu, *Chem. Eng. J.* **2023**, *454*, 140433.

[5] J. Zhang, X. Zhang, C. Xu, H. Yan, Y. Liu, J. Xu, H. Yu, L. Zhang, J. Shu, *Adv. Energy Mater.* **2022**, *12*, 2103998.

[6] C. Dai, L. Hu, H. Chen, X. Jin, Y. Han, Y. Wang, X. Li, X. Zhang, L. Song, M. Xu, H. Cheng, Y. Zhao, Z. Zhang, F. Liu, L. Qu, *Nat. Commun.* **2022**, *13*, 1863.

[7] Y. Wang, B. Wang, J. Zhang, D. Chao, J. Ni, L. Li, *Carbon Energy* **2023**, *5*, e261.

[8] Y. Wang, D. Chao, Z. Wang, J. Ni, L. Li, *ACS Nano* **2021**, *15*, 5420-5427.

[9] J. Zhang, Y. Wang, M. Yu, J. Ni, L. Li, *ACS Energy Lett.* **2022**, *7*, 1835-1841.

[10] Y. Sun, Y. Zhao, Q. Lei, W. Du, Z. Yao, W. Zhang, J. Si, Z. Ren, J. Chen, Y. Gao, W. Wen, R. Tai, X. Li, D. Zhu, *Adv. Mater.* **2023**, *35*, 2209322.

[11] F. Cui, R. Pan, L. Su, C. Zhu, H. Lin, R. Lian, R. Fu, G. Zhang, Z. Jiang, X. Hu, Y. Pan, S. Hou, F. Zhang, K. Zhu, Y. Dong, F. Xu, *Adv. Mater.* **2023**, *35*, 2306580.

[12] M. Lin, R. Qi, W. Zhang, Z. Ren, J. Si, Q. Lei, Y. Sun, H. Li, J. He, Q. Zhang, J. Zeng, W. Wen, Y. Gao, X. Li, D. Zhu, *Adv. Energy Mater.* **2024**, *14*, 2401288.

[13] S.-C. Wu, Y. Ai, Y.-Z. Chen, K. Wang, T.-Y. Yang, H.-J. Liao, T.-Y. Su, S.-Y. Tang, C.-W. Chen, D. C. Wu, Y.-C. Wang, A. Manikandan, Y.-C. Shih, L. Lee, Y.-L. Chueh, *ACS Appl. Mater. Interfaces* **2020**, *12*, 27064-27073.

[14] R. Zhou, Z. Hou, Q. Liu, X. Du, J. Huang, B. Zhang, *Adv. Funct. Mater.* **2022**, *32*, 2200929.
